# Metabolomic characterization of myocardial ischemia-reperfusion injury in ST-segment elevation myocardial infarction patients undergoing percutaneous coronary intervention

**DOI:** 10.1038/s41598-019-48227-9

**Published:** 2019-08-13

**Authors:** Arun Surendran, Michel Aliani, Amir Ravandi

**Affiliations:** 1 Cardiovascular Lipidomics Laboratory, St. Boniface Hospital, Albrechtsen Research Centre, University of Manitoba, Winnipeg, Canada; 2 Department of Human Nutritional Sciences, University of Manitoba, Winnipeg, Canada; 3 Department of Physiology and Pathophysiology, Faculty of Health Sciences, University of Manitoba, Winnipeg, Canada

**Keywords:** Translational research, Molecular medicine

## Abstract

Aim: The aim of the study was to discover the metabolomic changes in plasma that occur during human Ischemia-Reperfusion (I/R) injury and to evaluate the diagnostic utility of plasma metabolomic biomarkers for determination of myocardial injury. Deciphering the details of plasma metabolome in ST-segment elevation myocardial infarction (STEMI) patients before and after primary percutaneous coronary interventions (PPCI) would allow for better understanding of the mechanisms involved during acute myocardial ischemia and reperfusion in humans. We performed a detailed non-targeted metabolomic analysis of plasma from 27 STEMI patients who had undergone PPCI in the first 48 hrs employing a LC-MS approach. Plasma metabolome at ischemic condition was compared to multiple time points after PPCI which allowed us to focus on changes in the reperfusion phase. Classification of the differential metabolites based on chemical taxonomy identified a major role for lipids and lipid-derived molecules. Biochemical pathway analysis identified valine, leucine and isoleucine biosynthesis, vitamin B6 metabolism and glutathione metabolism as the most significant metabolic pathways representing early response to I/R injury. We also identified phenyl alanine, tyrosine, linoleic acid and glycerophospholipid metabolism as the most significant pathways representing late response to I/R injury. A panel of three metabolites pentadecanoic acid, linoleoyl carnitine and 1-linoleoylglycerophosphocholine was discovered to have diagnostic value in determining the extent of I/R injury based on cardiac biomarkers. Using a non-targeted LC-MS approach, we have successfully generated the most comprehensive data to date on significant changes in the plasma metabolome in STEMI patients who had undergone PPCI in the first 48 hrs showing that lipid metabolites represent the largest cohort of molecules undergoing significant change.

## Introduction

Despite considerable improvements in mortality rates over the past two decades, coronary artery disease (CAD) remains the leading cause of morbidity and mortality worldwide, with myocardial infarction (MI) a common manifestation of this disease^1^. After an acute myocardial infarction, early and successful myocardial reperfusion by means of primary percutaneous coronary interventions (PPCI) is the treatment of choice for limiting the size of myocardial infarction and improving clinical outcomes. But the process of rapid restoration of blood flow to myocardium (reperfusion) can lead to additional injury, a phenomenon known as myocardial ischemia/reperfusion (I/R) injury^2^. The largest impact of I/R in the clinical setting is during percutaneous coronary intervention of patients presenting with an occluded coronary artery known as STEMI^3^ (ST elevation myocardial infarction). Even with the best clinical care, the 30 day rate of Major Adverse Cardiac Events (MACE) for these patients is 10%^4^.

Although there have been extensive *in vitro* and *in vivo* studies on the concept of I/R injury, there has yet to be a therapeutic option available to minimize the harmful effects of reperfusion injury. Many studies have investigated a single molecule or a single pathway as a potential therapeutic avenue. As in many other pathological processes there are multiple pathways involved in reperfusion injury. The numerous advancements in “omics” technology platforms in recent years have allowed us to determine the changes at the genome and proteome level. However, only changes in the metabolite level will allow us to understand the downstream effects of perturbed cellular pathways^5^. The heart is the most metabolically demanding organ in the body and its metabolic perturbation leads to changes in the metabolome of body fluids including plasma^6^. Therefore changes in plasma metabolites may reflect underlying cardiac diseases progression. Previous studies have reported individual metabolic biomarkers for heart failure, myocardial infarction, and CAD^7–9^. However, there is little information available on the metabolomic changes in human plasma during I/R injury.

In this study we did a detailed non-targeted plasma metabolomic analysis employing a liquid chromatography coupled with mass spectrometry (LC-MS) platform. We investigated the time-effect changes in human plasma metabolome before and after PPCI during the first 48 hours in the setting of I/R injury in patients presenting with STEMI. We also identified a panel of plasma metabolite markers with potential for determining the extent of I/R injury.

## Materials and Methods

### Patients and study design

A total of 108 plasma samples from 27 patients presenting with STEMI enrolled from St. Boniface Hospital, Canada between June 2014 and July 2015 formed the study cohort. Both verbal and written consent were obtained from all subjects.

**Figure 1:**
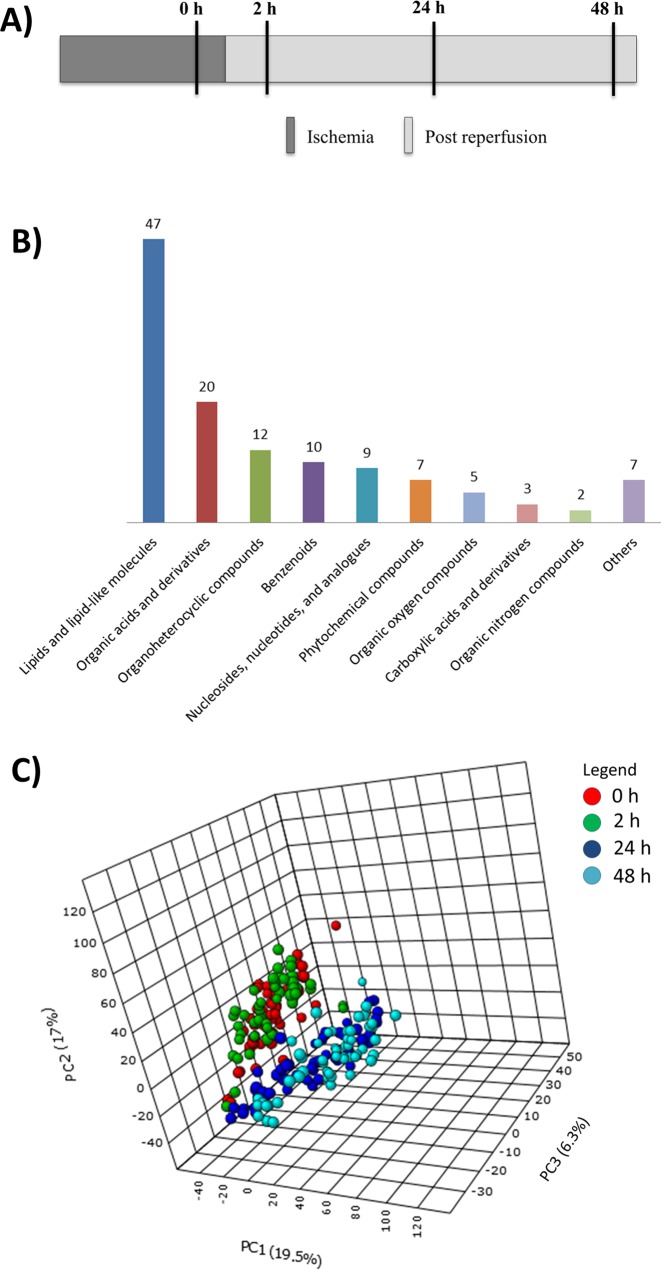
(**A**) Overall study design: The samples were collected by venipuncture at four different time intervals including the time of arrival at the cardiac catheterization laboratory for primary PCI (0 h ischemic condition (pre angioplasty)), 2 h post angioplasty (time-2), 24 h post angioplasty (time-3), and 48 h post angioplasty (time-4). (**B**) Metabolite classification based on chemical taxonomy: The number of metabolites from each metabolite super family of those identified (p < 0.001) in the analysis. (**C**) PCA plot of samples across different time intervals: PCA score plot showing a clear separation between samples from initial time points (0 h, 2 h) and final time points (24 h, 48 h). Also, it shows a close association among the samples of initial time points (0 h, 2 h) and final time points (24 h, 48 h).

Inclusion criteria were: ages between 18 and 75, confirmation of STE (ST segment elevation) on 12 lead ECG, presentation with chest pain and documentation of occluded coronary artery with coronary angiography. The overall study design is shown in Fig. 1A. The samples were collected by venipuncture at four different time intervals including the time of arrival at the cardiac catheterization laboratory for primary PCI (0 h, time-1), 2 h post angioplasty (time-2), 24 h post angioplasty (time-3), and 48 h post angioplasty (time-4). Blood samples were collected in EDTA- treated tubes and immediately centrifuged at 2500 × g for 10 minutes at 4 °C in a refrigerated centrifuge to harvest plasma. To avoid frequent retrieval of the aliquot box from the −80 °C freezer that can cause small scale thawing, exactly 100 microliters of plasma samples required for sample preparation was aliquoted in cryogenic vials, (Fisher Scientific, NY, USA) snap-frozen in liquid nitrogen and sealed under a stream of nitrogen gas and frozen at −80 °C prior to metabolite extraction. Average time of sample collection to plasma separation and aliquoting were less than 30 min. Approval for this study was obtained from both the University of Manitoba and the St. Boniface Hospital research ethics boards. Clinical data was reviewed retrospectively.

### Sample size calculation

To determine the necessary number of subjects needed to ensure the detection of a true statistical difference across different time intervals, we performed a power analysis (Supplementary Fig. 1). The power analysis indicated that we will have over 80% power to detect discriminating metabolites using approximately 25 subjects in each group. By considering the results from power analysis and effort required, we confined our study with 27 subjects. A detailed procedure for sample size consideration employed was provided in the Supplementary Data.

### Extraction of plasma metabolites

Extraction of low-molecular-weight (<1500 Da) metabolites in plasma samples was done by as previously described^10^. Briefly, a volume of 100 microliters of plasma was mixed with a volume of 200 microliters of acetonitrile. The sample was centrifuged and supernatant of the mixture was collected and then analysed. Each plasma sample was extracted in duplicate. Also, a quality control (QC) mixture made of pooled plasma samples were used to validate the extraction and LC-MS method^11^. More detail regarding blood sample collection, metabolite extraction and QC runs are provided in the Supplementary Data.

The metabolites were separated on a 1290 Infinity Agilent HPLC system from Agilent Technologies (CA, USA) using a Zorbax Extend-C18 analytical column (2.1 mm × 50 mm I.D., particle size 1.8 µm, Agilent Technologies, USA). Mass spectral analysis of eluting peptides from the analytical column was carried out on a 6538 UHD Accurate Q-TOF LC/MS from Agilent Technologies (CA, USA) controlled by MassHunter Workstation Software (v 7.0). All analyses were performed in both positive and negative mode ESI employing a dual ionization source. The acquired raw LC/MS data (‘.d files’) was preprocessed using Agilent MassHunter Qualitative Analysis (MHQ, vB.07) and Profinder (vB.06) software. Agilent Mass Profiler Professional (MPP, v12.6), MetaboAnalyst^12^ software v3.0 and v4.0 (McGill University, Quebec, Canada), MetScape software v3.0 (http://metscape.ncibi.org), and R statistical software v3.5.2 (https://www.r-project.org) were used for data processing and statistical analysis. In addition, receiver operating characteristic (ROC) analysis was used to evaluate the diagnostic capability of metabolites which can serve as potential biomarkers. Detailed description of LC conditions, MS parameters, compound identification, data processing and statistical analysis were provided in the Supplementary Data. Also, a summary of the metabolomic workflow was provided in Supplementary Table. 1.

## Results

Table 1 shows the demographic and laboratory data of the patients. We have also collected data on biochemical and cardiac markers including creatine kinase (CK) and high sensitivity troponin (TnT) for these patients. The mean age of our population was 61 years with 40% female and 15% had a diagnosis of type 2 diabetes. The prevalence of hypertension, dyslipidemia and smokers were 30%, 37% and 22% respectively and only a few (7%) had a previous history of known CAD. In our patients, 37% had occlusion in the left anterior descending (LAD) coronary artery, 48% in the right coronary artery (RCA) and 19% in the circumflex coronary artery. Based on patient’s characteristics, our study cohort is similar to previous STEMI studies as shown by patients in the FAST-MI Program (French Registry of Acute ST-Elevation or Non-ST-elevation Myocardial Infarction)^13^.

**Table 1:** Demographic and laboratory characteristics of STEMI patients.

Characteristics (n = 27)	Results
**Age, yrs**.	61.55 ± 14.51
**Female (%)**	44
**LVEF**	59.29 ± 13.07
**Body mass index, kg/m** ^**2**^	28.48 ± 5.08
**Comorbidity (%)**	
Hypertension (%)	30
Diabetes mellitus (%)	15
Current smoker (%)	22
Dyslipidemia (%)	37
Hx of CAD (%)	7
**Laboratory data**	
TG, mmol/l	1.9 ± 1.65
TC, mmol/l	4.69 ± 1.27
HDL-C, mmol/l	1.14 ± 0.33
LDL-C, mmol/l	2.84 ± 1.06
CR, mmol/l	92.33 ± 43.97
Additional parameters*	
Minutes from onset of chest pain to reperfusion	140 [50–360]
Peak CK (Units/L)	1017 [136–7028]
Peak TnT (ng/L)	1862 [503–10000]
**Culprit vessel (%)**	
LAD Infarct (%)	37
RCA Infarct (%)	48
Circumflex Infarct (%)	19
**Medications at baseline (%)**	
ASA	100
Intravenous heparin	100
ACEI/ARB	15
Beta blocker	7
Statin	15
Ticagrelor	96
Clopidorgrel	4

Values are mean ± SD or percent of patients otherwise specified.

*Median [Range].

GP IIb/IIIa inhibitors were not used in this cohort.

LVEF = left ventricular ejection fraction; Hx of CAD = history of coronary artery disease; TG = triglyceride; TC = total cholesterol; HDL-C = high-density lipoprotein cholesterol; LDL-C = low-density lipoprotein cholesterol; CR = creatinine; CK = creatine kinase; TnT = troponin; LAD = Left Anterior Descending coronary artery; RCA = Right coronary artery; ASA = Acetylsalicylic acid; ACEI = Angiotensin-converting enzyme (ACE) inhibitors; ARB = Angiotensin II receptor blockers.

Utilizing “Find by Formula” (FBF) algorithm, by searching against the database, resulted in the identification of 765 and 670 compounds in positive and negative modes, respectively in plasma from patients presenting with STEMI undergoing PPCI. The endogenously present metabolites which are detected and quantified in blood from Human Metabolome Database^14^ (HMDB) served as the database. We enlarged the scope of this database by adding further 350 metabolites from published literature known to be associated with cardiac diseases. In order to find missing features and to give higher confident identifications by further minimizing the false positives and false negatives identifications, batch recursive analysis was done on the already identified compounds. Subsequent to recursive analysis, the list was further reduced to 69 and 82 compounds in positive and negative modes, respectively. After adjusting for p-value after ‘Bonferroni FWER’ correction, only those feature satisfying p < 0.001 were considered as significantly differential metabolites across all the four time intervals. The final annotated list contained 130 significantly differential metabolites (p < 0.001) across all the four time intervals. Of these, 55 elements compounds were identified exclusively in “Positive” ESI mode, 64 compounds were identified exclusively in “Negative” ESI mode and 11 compounds were identified in both the modes (Supplementary Fig. 2 and Supplementary Table 2). A total of 59 metabolites from this list were already recognized metabolic signatures of ischemia, myocardial infarction or other forms of CAD including non-obstructive coronary atherosclerosis, stable angina pectoris or unstable angina pectoris. The details of the significant compounds with their associated pathways and respective references from literature are shown in Supplementary Table 3.

### Taxonomy of significant metabolites

Classification of significant metabolites (p < 0.001) based on chemical taxonomy showed that lipids and lipid-derived molecules (38%) formed the major constituents of the altered metabolomic profile followed by organic acids, organo heterocyclic compounds, benzenoids, nucleotides and phytochemical compounds (Fig. 1B). These represent compounds with significant change amongst the four time points.

### Changes in metabolic profile before and after the reperfusion

**Figure 2:**
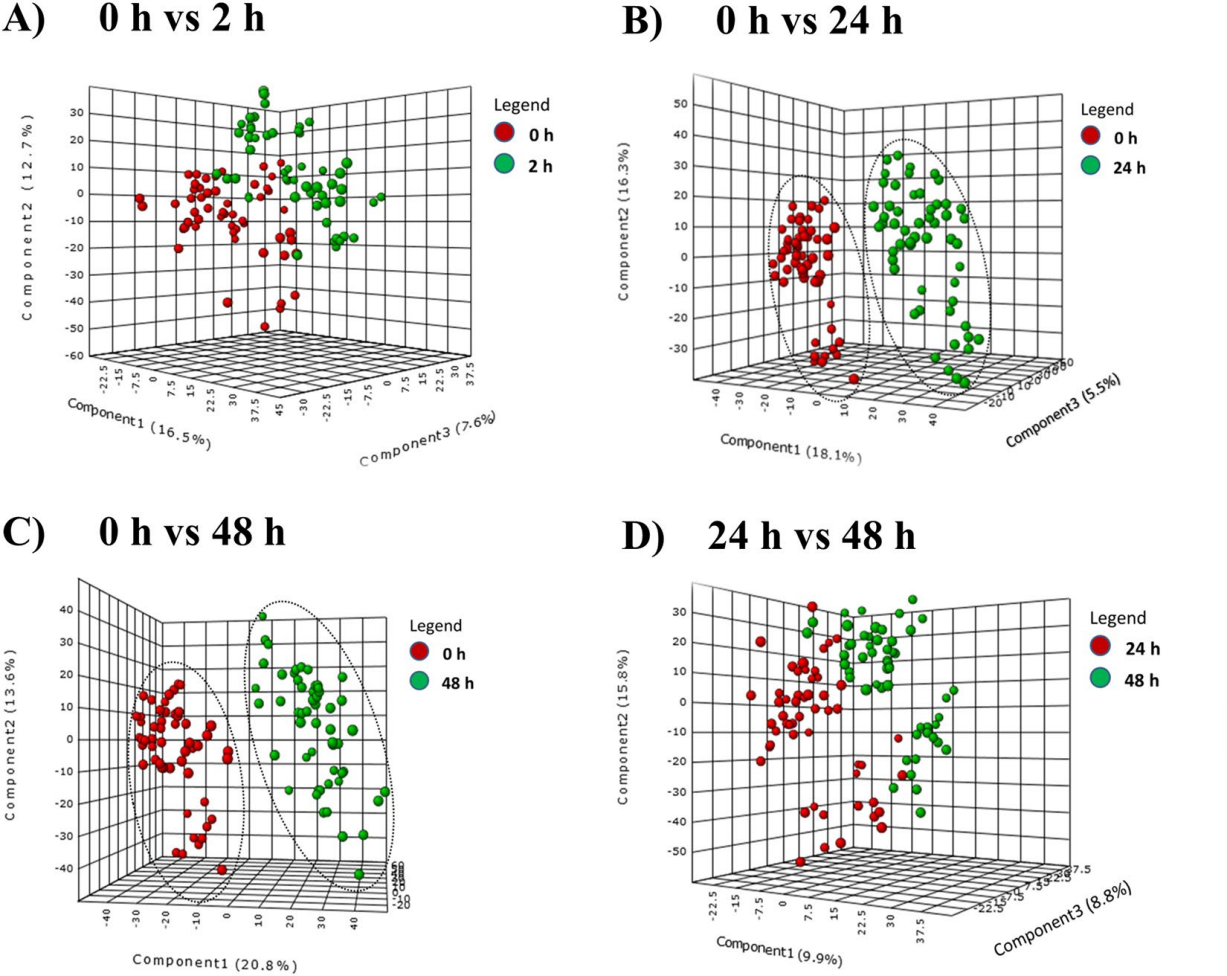
PLS-DA scores plots for comparison of the metabolic profiles: The partial least squares analysis (PLS-DA) score plots compared **(A)** ‘0 h ischemic’ time point to ‘2 h post reperfusion’ time point **(B)** ‘0 h ischemic’ time point to ‘24 h post reperfusion’ time point, **(C**) ‘0 h ischemic’ time point to ‘48 h post reperfusion’ time point and **(D)** ‘24 h post reperfusion’ time point to ‘48 h post reperfusion’ time point. The different colours red and green correspond to different time points.

To understand the changes in plasma metabolome before and after the PPCI, we employed multivariate pattern recognition tools such as principal component analysis (PCA) and partial least squares-discriminant analysis (PLS-DA). These visualizing plots were built based on the metabolite concentrations of 130 differential metabolites at each time interval. The PCA plot (Fig. 1C) revealed that not only there was a clear separation in metabolic profile between initial time intervals (0 h, 2 h) and final time intervals (24 h, 48 h) but also, there was a close association existed among the initial time intervals (0 h, 2 h) and final time intervals (24 h, 48 h). In line with the PCA plot, PLS-DA plot (Fig. 2) also confirmed a clear separation between ‘24 h post reperfusion’ time point and baseline (0 h, ischemic condition) (Fig. 2B) and a tight clustering between the ‘2 h post reperfusion’ time point and baseline (Fig. 2A).

### Early and late response to reperfusion

**Figure 3:**
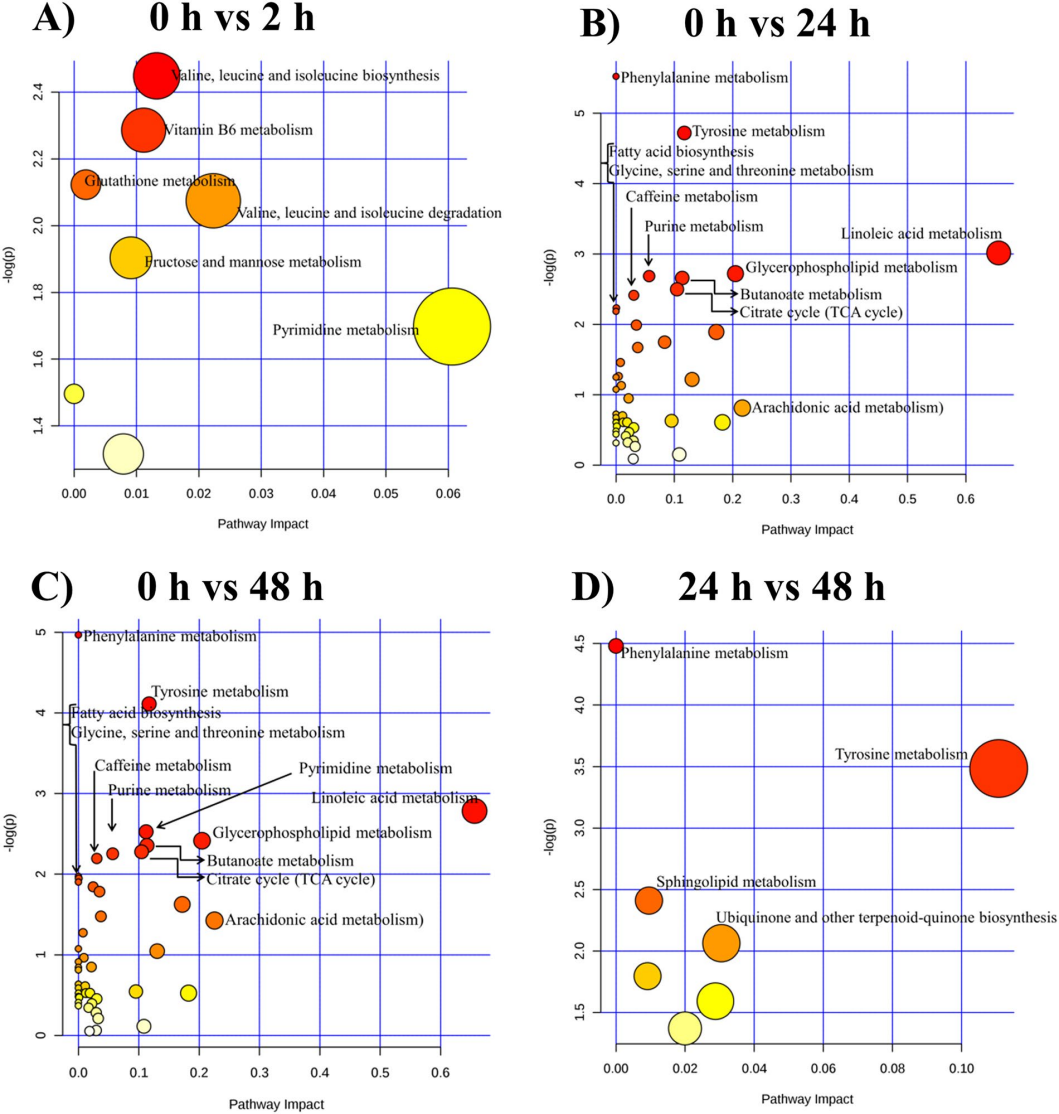
MetPA analysis of metabolic changes: TheMetPA analysis shows metabolic pathways of differential metabolites by comparing **(A)** ‘0 h ischemic’ time point to ‘2 h post reperfusion’ time point **(B)** ‘0 h ischemic’ time point to ‘24 h post reperfusion’ time point, (**C**) ‘0 h ischemic’ time point to ‘48 h post reperfusion’ time point and **(D)** ‘24 h post reperfusion’ time point to ‘48 h post reperfusion’ time point. The size and color of each circle indicate the significance of the pathway ranked by p-value (red: higher p-values and yellow: lower p-values) and pathway impact score (the larger the circle the higher the impact score), respectively.

Metabolites showing significant changes were analyzed using Metabolomics Pathway Analysis^15^ (MetPA) analysis to understand the metabolic pathways that were impacted due to primary PCI (Fig. 3). Based on impact scores, the most significant pathways (FDR <= 1) representing early response to reperfusion immediately after PPCI (2 h after reperfusion) (Fig. 3A) were found to be associated with valine, leucine and isoleucine biosynthesis, vitamin B6 metabolism and glutathione metabolism. Likewise, the most significant pathways (FDR < 1) after PPCI (24 h and 48 h after reperfusion) representing late response to reperfusion were found to be associated with the metabolism of following compounds: (1) phenyl alanine, (2) tyrosine, (3) linoleic acid and (4) glycerophospholipid (Fig. 3B,C). The details of metabolites involved at each time point were shown in Supplementary Table 4 and Supplementary Fig. 3.

### Highly correlated metabolites

**Figure 4:**
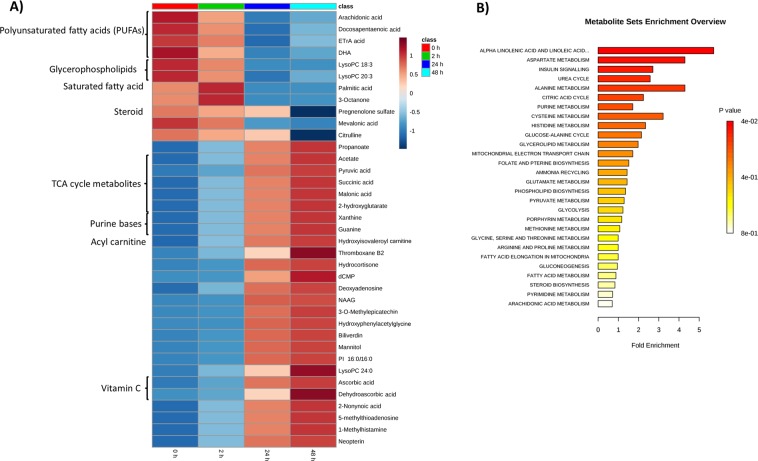
(**A**) Heat map of significant metabolites obtained from correlation analysis: The heat map constructed with highly correlated metabolites (n = 37), |r| > 0.9. The colors ranging from blue to orange indicates more concentration of metabolites. ETrA acid = 5,8,11-Eicosatrienoic acid; DHA = docosahexaenoic acid; NAAG = N-Acetylaspartylglutamic acid; LysoPC = lysophosphatidylcholine; PI = phosphatidylinositol; TCA = tricarboxylic acid. (**B**) Summary plot of metabolite set enrichment analysis (MSEA) of highly correlated metabolites: The horizontal bar graph summarizes the metabolic pathways that were significantly enriched by this group of functionally related metabolites (n = 37) during the setting of I/R injury. The color code corresponds to the calculated p-values (red: p = 4 × 10^−2^ to white: p = 8 × 10^−1^).

**Figure 5:**
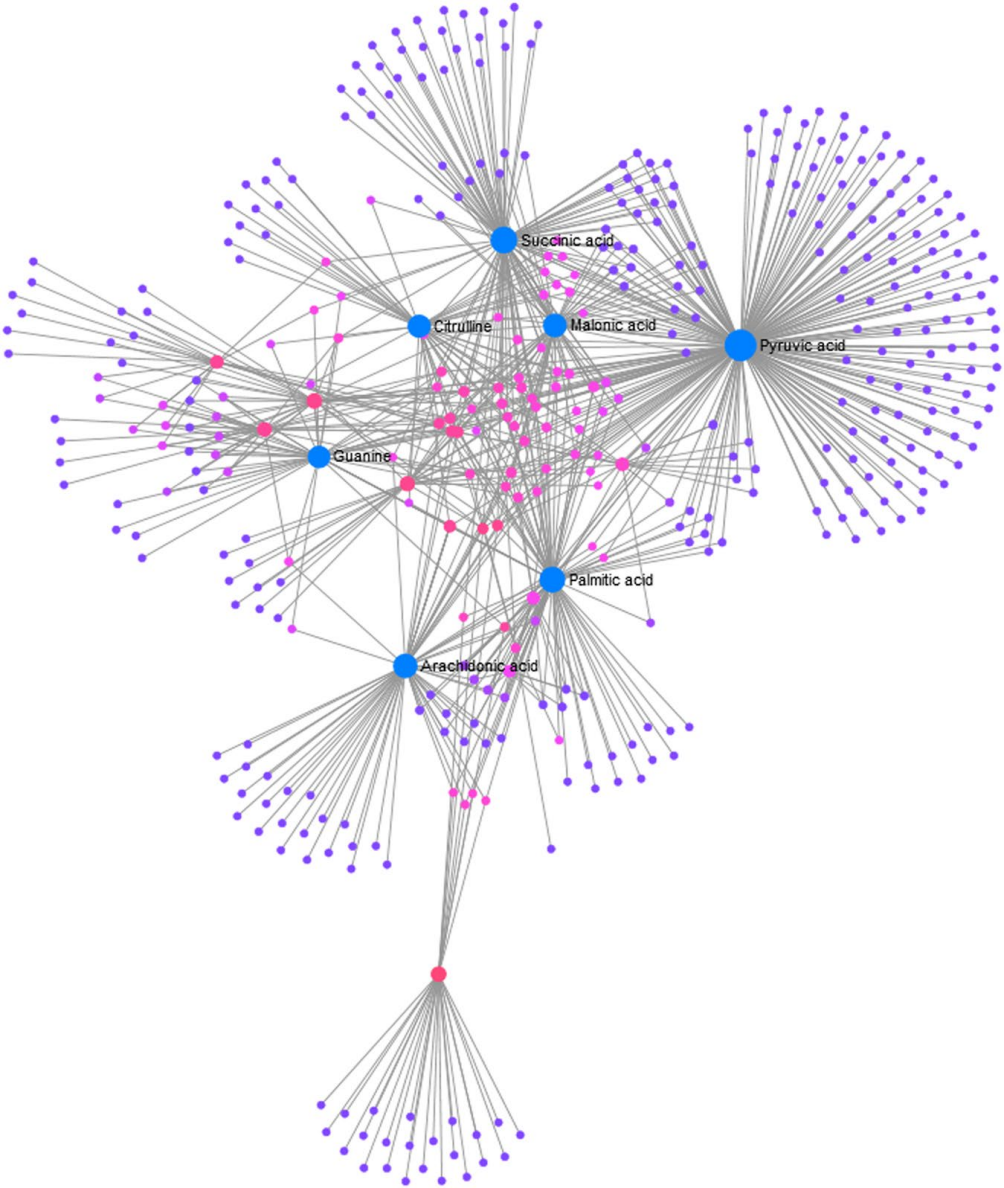
Network plot highlighting the highly correlated metabolites: The nodes represent metabolites and edges represent biochemical reactions. Only significant (|r| > 0.9) correlations are drawn. The blue nodes represent the most significant hub nodes in establishing connection between the sub-networks in the flow of information. Pyruvic acid, Succinic acid, Malonic acid, Palmitic acid and Arachidonic acid constitute the most significant sub nodes.

**Figure 6:**
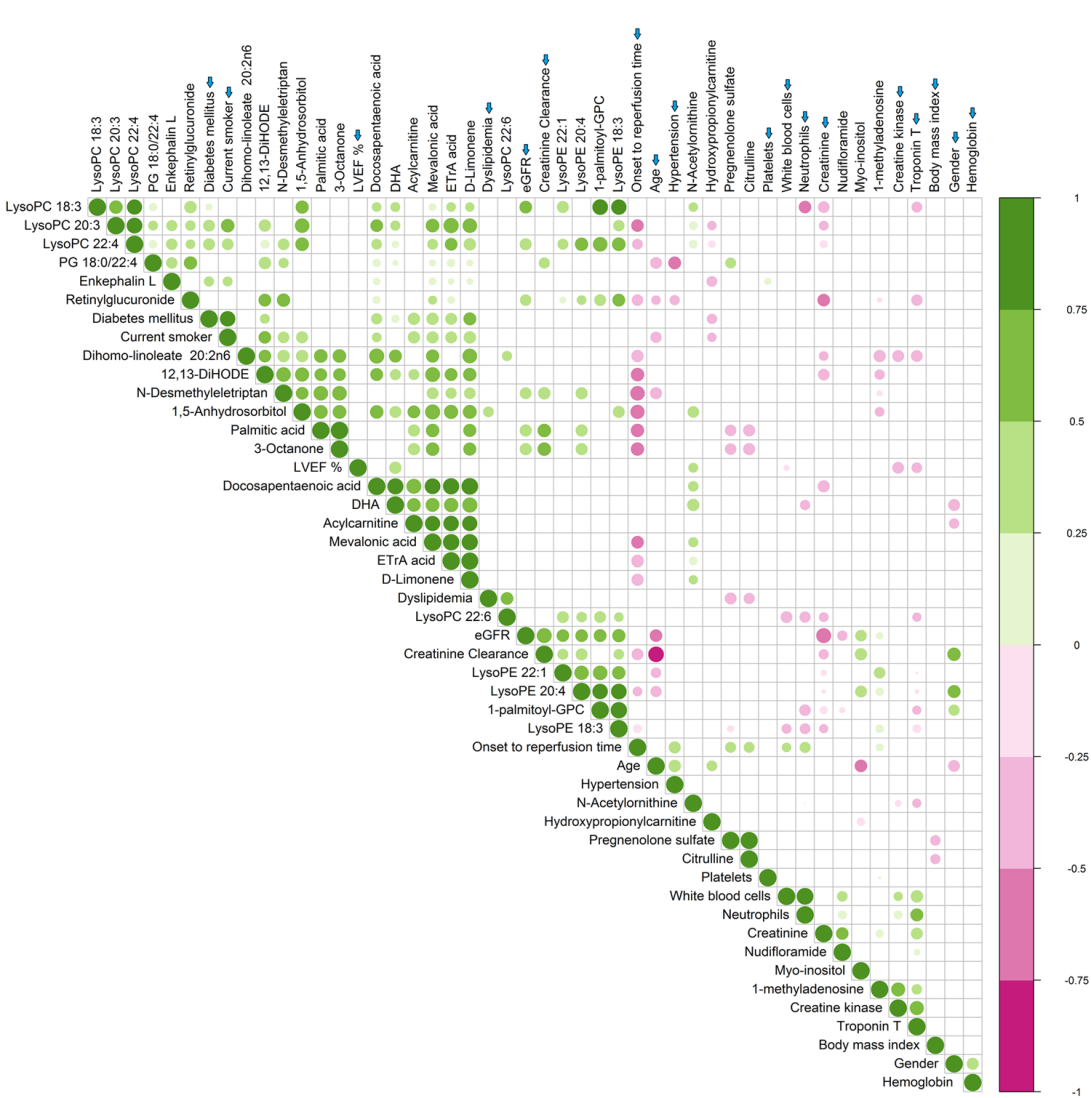
Correlation between clinical parameters and plasma metabolites at baseline: The figure shows the correlation matrix ordered by hierarchical clustering between the important clinical parameters and plasma metabolites at baseline (0 h, time-1). Positive correlations are displayed in green; negative correlations are displayed in pink; blue arrow indicates the clinical factors. Color intensity and the size of the circle are proportional to the correlation coefficients. In the right side of the correlogram, the legend color shows the correlation coefficients and the corresponding colors. Correlations with p-value > 0.05 as computed by Spearman correlations were considered as insignificant and were left blank. Only those metabolites factors (30 metabolites) having more than two significant correlations (p < 0.05) with any of the clinical parameters were used to construct the correlogram. ETrA acid = 5,8,11-Eicosatrienoic acid; DHA = docosahexaenoic acid; LVEF = left ventricular ejection fraction; 1-palmitoyl –GPC = 1-palmitoyl glycerophosphocholine.

Finding the highly correlated metabolites in the metabolite set becomes imperative as most of the high correlations may be due to either (1) stronger mutual control by a single enzyme or (2) variation of a single enzyme level much above others^16^. This can also help unravel the biological basis of underlying phenotypic or disease conditions^17,18^. To envisage this metabolite correlation, a heat map (Fig. 4A) was made with only highly correlated metabolites (n = 37) amongst 4 time points based on Pearson correlation (coefficient value, |r| > 0.9). This heat map shows the relative concentration of 37 highly correlated metabolites across different time intervals. To identify subtle but substantial changes among these correlated compounds, Metabolite Set Enrichment Analysis (MSEA) was performed on these functionally related metabolites (n = 37) along with their relative concentrations by using the web-based platform MetaboAnalyst (Fig. 4B). The pathways significantly enriched by these related compounds were (FDR <= 1) were (1) linoleic acid and alpha linolenic acid metabolism, (2) aspartate metabolism, (3) insulin signaling, (4) urea cycle, (5) alanine metabolism and (6) citric acid cycle. A correlation network plot showing connectivity and information flow was depicted in Fig. 5. This metabolic network plot depicts the connection between highly correlated metabolites based on STITCH (‘search tool for interactions of chemicals’)^19^ database, such that only highly confident interactions are shown. In this metabolite – metabolite network presentation, the most significant ‘hub nodes’ or metabolites that were involved in the flow of information between the different pathways were pyruvic acid, succinic acid, malonic acid, palmitic acid and arachidonic acid. To further investigate the relationship between the plasma metabolites and important clinical factors at baseline (0 h, time-1), correlations were also calculated for all pairs of metabolite-clinical factors using the R statistical package ‘corrplot’. The resulting correlation plot (Fig. 6) was presented as a clustered matrix in which rows and columns are ordered such that correlated variables are close to each other. At baseline (Fig. 6), the plasma concentrations of Lyso PE (18:3), Lyso PE (20:4), Lyso PE (22:1) showed a strong positive correlation with Glomerular Filtration Rate (eGFR), as were a number of other metabolites. Likewise, the plasma concentrations of 3-Octanone, palmitic acid, 12,13 DiHODE and few other metabolites were negatively correlated with duration of ischemia since onset of chest pain to reperfusion. In the full dataset, though there are a few clusters of correlated metabolites and clinical parameters, the overall correlation between the metabolites and clinical parameters is comparably weak. As expected, we can see a significant positive correlation (Supplementary Fig. 4) between ischemic time (duration from onset of chest pain to reperfusion) and the level of troponin in our cohort.

### Metabolites and clinical biomarkers of cardiac cell death

**Figure 7:**
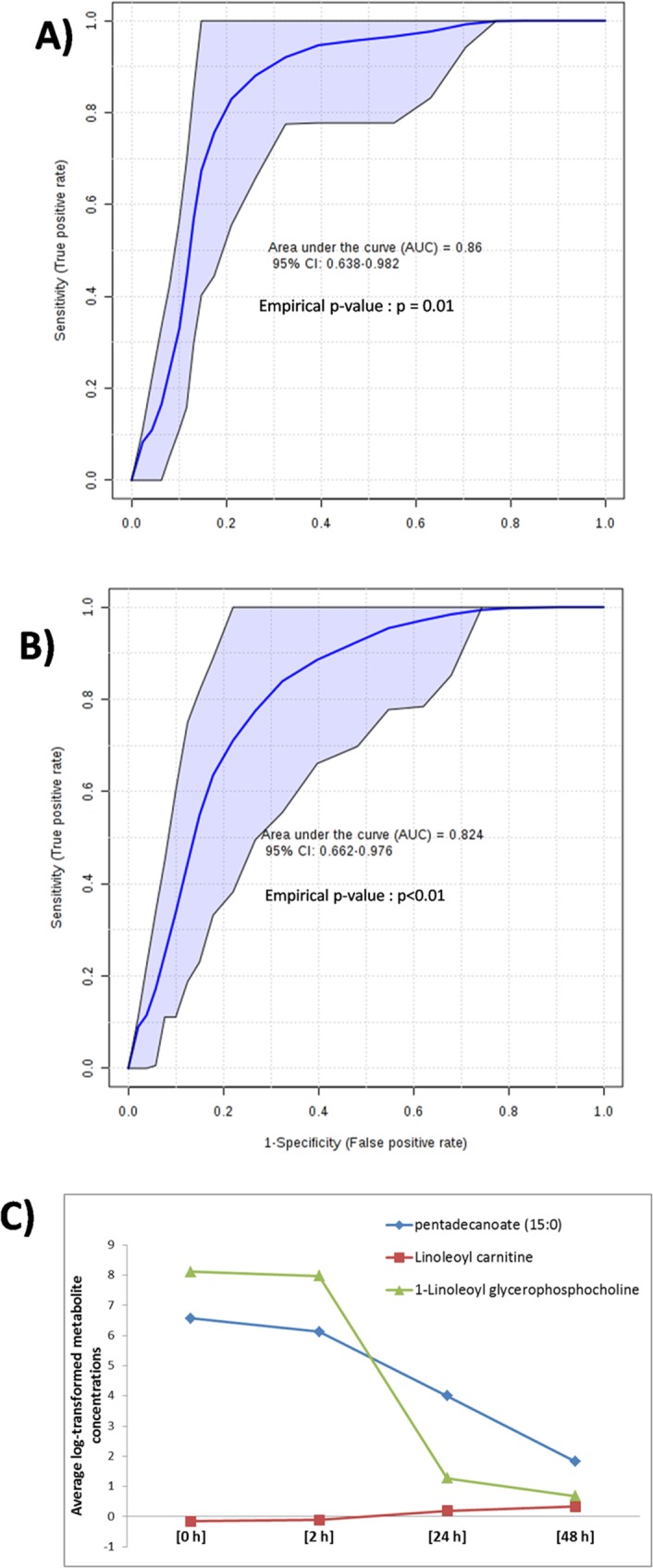
Multivariate ROC plots based on the troponin and creatine kinase (CK) concentration. (**A**) Receiver operating characteristic (ROC) plot obtained by combining three metabolites namely pentadecanoic acid, linoleoyl carnitine and 1-linoleoylglycerophosphocholine. The curve is based on the median troponin value of the cohort (Peak TnT) and is represented by an area under the curve (AUC) of 0.86 indicating good predictive ability. (**B**) The multivariate ROC curve discriminates individuals based on the median creatine kinase value (Peak CK). The curve is represented by area under the curve (AUC) of 0.82 indicating good predictive ability. The empirical p-value (p-value < 0.05) shows the statistical significance of these biomarker models. (**C**) The line plot shows the relative concentration of three potential metabolic biomarkers across different time intervals.

To find the links between changes in metabolite concentrations in plasma with STEMI patient physiology/pathology, we sought to find metabolites that are closely related to myocardial cell death and clinical outcomes. For that plasma metabolites were further evaluated for predictive accuracy for discriminating large infarct size patients from small infarct size patients based on troponin^20^ and creatine kinase^21^ (CK) concentrations which are the ‘gold standard’ biomarkers of cardiac cell death. The sample population was classified into two groups as ‘*Above median* Troponin’ group and ‘*Below median* Troponin’ group centered on the median troponin value (peak TnT, 1862 ng/L) of the cohort. We manually selected a combination of three metabolites namely pentadecanoic acid, 1-linoleoylglycerophosphocholine and linoleoyl carnitine to create a biomarker model to distinguish between these two groups using RF (random forest) algorithm^22^. This biomarker model was built using the metabolic profile at the time of admission. In order to produce a smooth ROC curve, 100 cross validations (CV) were performed and the results were averaged to generate the plot (Fig. 7A).From the ROC plot, it was evident that the combination of these 3 metabolites was a good classifier with an area under the curve (AUC) equal to 86%. The sample population was again classified into two groups centred on median CK value (peak CK, 1017 Units/L) of the cohort. The resulting ROC plot (Fig. 7B) with an AUC value of 82%, further reinforced the diagnostic ability of these 3 metabolites to serve as potential biomarkers in determining the impact of myocardial injury. To deal with the problem of over-fitting and to assess the statistical significance of our biomarker model with this relatively smaller sample size, permutation tests were performed using 100 cross-validations on the metabolite data. The resulting empirical p-value obtained (p-value < 0.05) shows the statistical significance of this biomarker model.

## Discussion

The main objective of our study was to characterize the altered metabolic pathway(s) in the first 48 hours after primary PCI which represent the reperfusion phase. To our knowledge, our study is the most comprehensive metabolomic analysis of human plasma ever undertaken during the first 48 hrs in the setting myocardial I/R injury in patients presenting with STEMI. In this study, we employed a repeated-measure research design by doing serial blood sampling from all STEMI patients both pre and post angioplasty. In repeated measures designs, each subject serves as their own biological control^26^ thereby reducing the intra patient variability. This allows the analysis to focus precisely on intervention effects and allows for fewer subjects to detect a desired effect size^27^ with increased statistical power.

A handful of metabolomic studies have been conducted in patients presenting with STEMI^23–25^. But none of these studies have investigated the time-effect changes in plasma metabolome before and after PPCI which is crucial to provide insights into the altered metabolic pathways with clinical relevance during I/R injury.

Our study had three major findings. Firstly, we identified a total of 130 plasma metabolites (Supplementary Table. 3) which were significantly (p < 0.001) affected prior to coronary intervention and in the follow up time intervals in the setting myocardial I/R injury as outlined. Secondly, we used metabolic profiling to identify early and late response in plasma metabolome in response to I/R injury. Notably, linoleic acid and alpha linolenic acid metabolism pathway represent the most significant change in plasma metabolome among the related metabolites. Thirdly, classification using ROC analysis identified pentadecanoic acid, linoleoyl carnitine and 1-linoleoylglycerophosphocholine as the top discriminating metabolites in determining the extent of myocardial injury.

The lipids and lipid-derived molecules formed the major components (38%) of the altered metabolomic profile (Fig. 1B) in the setting of I/R injury during the first 48 hours. Our previous work on *in vivo* models of I/R injury have also shown that during reperfusion, there are significant changes within bioactive lipid molecules^28,29^. The multivariate PCA and PLS-DA plots (Figs 1C and 2), showed that the underlying plasma metabolomic change was progressive after 24 hours post primary PCI, with a comprehensive change in the plasma metabolome compared to baseline (0 h, ischemic condition).

The pathway impact analysis revealed key metabolites and perturbed pathways (Fig. 3) that shed light on the early and late changes in plasma metabolome during I/R injury. Immediately post PPCI (Fig. 3A), when compared to pre PPCI, metabolic pathways for valine, leucine and isoleucine biosynthesis, vitamin B6 metabolism and glutathione metabolism were observed to have significant change. Among the metabolites in these altered pathways, the three intermediaries of glutathione metabolism namely glutathione, oxidized (GSSG), ascorbic acid and dehydroascorbic acid are of significant importance in the setting of myocardial reperfusion injury^30^. The extent of myocardial injury sustained during reperfusion is very dependent on the effectiveness of its antioxidant defenses^31^. Compared to ischemic period (0 h), there were marked increase in levels of ascorbic acid and oxidized forms of ascorbate and glutathione namely dehydroascorbic acid and GSSG, following reperfusion. This suggests that these tissue hydrophilic antioxidants may be the first line of antioxidant defences to curb the generation of reactive oxygen species (ROS) following reperfusion^31^. The most significant pathways (Fig. 3B,C) representing late response in plasma metabolism at 24 h and 48 h post PPCI were phenylalanine metabolism, tyrosine metabolism, linoleic acid metabolism and glycerophospholipid metabolism which were different from those seen immediately post PPCI.

Measures of correlation between metabolites in replicate profiles can be very informative about the underlying biological system^16^. From the heat map (Fig. 4A) constructed with highly correlated metabolites (n = 37), it was evident that the concentration levels of certain free fatty acids (FFA) such as arachidonic acid (AA), docosapentaenoic acid (DPA), eicosatrienoic acid, docosahexaenoic acid (DHA), which are intermediaries of linoleic acid and alpha linolenic acid metabolism, and certain lysophospholipids such as lysoPC (18:3), lysoPC (20:3) were elevated at ischemic time point (0 h, pre angioplasty), but decreased progressively following reperfusion. The enzyme phospholipases A_2_ (PLA_2_) is known to play an important role in the hydrolysis of phospholipids^32^ especially phosphatidylcholines (PC), which leads to accumulation of FFA including (non-esterified) AA and DHA^33^. This non-esterified AA is then rapidly esterified to available lysophospholipids, or is converted into bioactive arachidonic acid metabolites, i.e., eicosanoids via cyclooxygenase (COX), lipoxygenase (LOX), or cytochrome P450 (CYP) epoxygenase enzymes^34^. From our study, the observed progressive decrease in the concentration levels of AA, DHA, DPA, lysophospholipids and their downstream products such as eicosatrienoic acid after reperfusion strongly endorses the possible part that PLA_2_ plays in disturbed phospholipid homeostasis during the transition from reversible to irreversible ischemic myocardial injury. This evidence also underlines the current understanding that lipoprotein-associated PLA_2_ is a significant predictor of cardiovascular outcome independent of traditional clinical risk factors^35^. In addition, few other metabolites like citrulline (a participant in urea cycle), mevalonic acid, 3-octanone and pregnenolone sulfate also exhibited the same co-variation (initially increases with subsequent decline) as the above metabolites.

Amongst highly correlated metabolites, several metabolites exhibited a pattern (Fig. 4A) which is different from the metabolites discussed above. The abundance levels of these metabolites declined at ischemic condition (0 h) compared to post reperfusion time intervals, but elevated noticeably following reperfusion. These include pyruvate, succinate, malonic acid, 2-hydroxyglutarate, acetate and propanoate which are known to involve directly or indirectly with TCA cycle metabolism. The variation in the metabolic concentrations of these compounds before and after reperfusion suggests an impaired TCA cycle metabolism and subsequent energy metabolism in the setting of I/R injury. The heat map (Fig. 4A) also shows that the concentration levels of certain other highly correlated metabolites, like deoxyadenosine, N-acetylaspartylglutamic acid (NAAG), 3-O-methylepicatechin, 2-nonynoic acid, 5-methylthioadenosine, 1-methylhistamine and neopterin also exhibited the same co-variation (initially low, but finally high) as the above metabolites.

Previous studies have shown that elevations in troponin (Troponin T) and creatine kinase (CK) or its specific MB (CKMB) isoform after primary PCI represent larger infarct size and are clearly associated with increased early and late mortality^36–38^. In our study cohort, a combination of three metabolites namely pentadecanoic acid (15:0), linoleoyl carnitine (18:2 carnitine) and 1-linoleoylglycerophosphocholine (18:2 lysoPC) exhibited good separating capability in discriminating small and large infarct size patients with an AUC value of 86 based on peak troponin concentration (Fig. 7A) and with an AUC value of 82 (Fig. 7B) based on peak CK concentration. The Fig. 7C shows the relative concentration of these three molecules across the four time points. For pentadecanoic acid and 18:2 lysoPC, their amount is high before reperfusion and their amount decreases considerably 2 h post reperfusion. In the case of 18:2 carnitine, its amount is negligible before reperfusion, but increases progressively post reperfusion. Thus, our data suggest that determining the concentration level of these three metabolites at the time of admission is a good indicator of myocardial infarct size following coronary angioplasty and subsequent increased late morbidity and mortality.

Until recently it was assumed that the existence of straight chain odd number fatty acids such as pentadecanoic acid (15:0) in normal physiological conditions were rare. Recent analytical instrumentation has proven this to be a misconception, since the presence of odd number carbon fatty acids such as 15:0 and 17:0 have been shown to occur as minor constituents in practically every natural fat and carry out many roles like synthesis of very-long-chain odd-numbered fatty acids, replenish the TCA cycle with anaplerotic intermediates and, hence, improve mitochondrial energy metabolism in nature^39^. But whether one or all of these known metabolic roles of 15:0 has a link to CVD risk is still debated. Though certain studies have reported the association of 15:0 with greater risk of CVD^40^, many studies like Elwood *et al*.^41^ showed that, in relation to coronary artery disease, it is inappropriate to accept an estimate of CVD risk based on plasma concentrations of 15:0 alone. Also, earlier studies have reported that lower serum levels of 18:2 lysoPC, a downstream product of lysophosphatidylcholine (LPC), has a tight relationship with increased arterial stiffness, increased resting heart rate and occurrence of silent myocardial ischemia^42,43^. Linoleoyl carnitine is a long-chain acyl fatty acid derivative ester of carnitine. Ischemic condition inhibits beta oxidation of fatty acids and leads to accumulation of toxic intermediates of beta oxidation, particularly long-chain acyl carnitine compounds like linoleoyl carnitine. These compounds are detrimental to cellular function and are known to enhance myocardial injury by inducing structural damage in ischemic myocytes^44,45^. From our study these three molecules were proved to be promising molecular markers for the determination of myocardial injury. To our knowledge, this is the first published report of a blood-based biomarker panel for the prediction of I/R injury following coronary intervention in patients presenting with STEMI. This has the capacity to identify novel pathways impaired during reperfusion injury and identify patients at high risk for myocardial damage after PPCI allowing for development of therapeutic intervention to minimize I/R injury.

## Study Limitations

Our study has several potential limitations that should be considered. First, though serial sampling performed in all STEMI patients both before and after PPCI helped minimize inter-individual variability and clinical confounders such as diet, drug effects, age, sex, and other co morbidities, our study population was comparatively small to identify metabolites that failed to reach minimal significance. But these metabolites which fail to reach minimum significance might be scientifically relevant. So it is important to adequately validate the identified metabolites in a relatively larger patient cohort. Second, though ROC analysis with 100 CV exhibited good prediction accuracy (better than 80%); these findings are exploratory in nature and should be confirmed in an independent patient cohort. Moreover, as with any untargeted metabolomic study, samples were analysed without authentic standards. This calls for further confirmation of these identified metabolites by employing a targeted metabolomic analysis.

## Conclusions

Using a non-targeted LC-QTOF-MS methodology, we have successfully generated the most comprehensive data to date on significant changes in the plasma metabolome in STEMI patients undergoing PPCI. The role of lipids and lipid metabolism pathways in the pathogenesis of atherosclerosis is already well understood. But there is little information about their role in reperfusion and I/R injury. From our study, we elucidated that lipid metabolism in general and phospholipid, linoleic acid and alpha linolenic acid metabolism in particular represent the largest change in the plasma metabolome post PPCI. We also identified a panel of three metabolites namely pentadecanoic acid, linoleoyl carnitine and 1-linoleoylglycerophosphocholine that could serve as plasma biomarkers in determining the extent of myocardial injury after PPCI. This knowledge could help to predict the response to PPCI and how to limit complications in STEMI patients after reperfusion. Given that there is currently no therapy available for I/R injury, we consider our results as a major step toward moving us closer to our ultimate goal of developing therapies to prevent myocardial reperfusion injury and improve clinical outcomes in patients with STEMI.

## Supplementary information

Supplementary information

Table 4

## Data Availability

Data and associated protocols are available to readers.
