# Supplementary material for: Metabolomic characterization of myocardial ischemia-reperfusion injury in ST-segment elevation myocardial infarction patients undergoing percutaneous coronary intervention
Source: Sci Rep. 2019 Aug 13;9:11742. doi: 10.1038/s41598-019-48227-9 (PMC6692400; doi:10.1038/s41598-019-48227-9)
Supplement: Supplementary file 1 — Supplementary information [file 41598_2019_48227_MOESM1_ESM.docx]

**Metabolomic characterization of myocardial ischemia-reperfusion injury in ST-segment elevation myocardial infarction patients undergoing percutaneous coronary intervention**

Arun Surendran^1, 3^, Michel Aliani^2, 3#^ and Amir Ravandi^1, 3#^

Cardiovascular Lipidomics Laboratory, St. Boniface Hospital, Albrechtsen Research Centre^1^, Department of Human Nutritional Sciences, University of Manitoba^2^, Department of Physiology and Pathophysiology, Faculty of Health Sciences, University of Manitoba^3^, Winnipeg, Canada

**Disclosures**

The authors declare that they have no competing interests.

**Corresponding authors^#^:**

Michel Aliani PhD

Department of Human Nutritional Sciences, University of Manitoba,

Albrechtsen Research Centre, Winnipeg, Manitoba, Canada, R2H 2A6.
R4024, 351 Taché Ave.
Winnipeg, MB, R2H 2A6 Canada

michel.aliani@umanitoba.ca

Amir Ravandi MD PhD, FRCPC,

Albrechtsen Research Centre

Y3508, Bergen Cardiac Care Centre, 409 Tache Avenue,

Winnipeg, Manitoba, Canada, R2H 2A6.

aravandi@sbgh.mb.ca

**SUPPLEMENTARY APPENDIX**

1. **MATERIALS AND METHODS**

**1.1. Sample size calculation**

Though sample size determination is essential, the metabolic phenotyping which are usually characterized by high dimensions with hundreds of features limit the use of conventional techniques developed for other omics sciences like proteomics and genomics. Since our goal is to identify discriminating metabolites across the time course of ischemia/reperfusion injury, the effect size between the time points mainly determines the sample size. In our study, the largest effect (n=107, differential metabolites) was observed between 0 h and 48 h time points, and we chose those two time points to calculate the statistical power. The power analysis was done using MetaboAnalyst software v3.0. The MetaboAnalyst does power calculation based on two assumptions: 1) the effect is indeed present in the data, and 2) the test statistic follows a normal or near normal (Students’t) distribution. A false discovery rate (FDR) of 0.1 was chosen as the significance criterion. The result (**Supplementary Figure.1**) indicated that power in our samples reaches an acceptable level (0.8) at a sample size of approximately 25.

**Supplementary Figure.1 Power vs. sample size, FDR = 0.1**


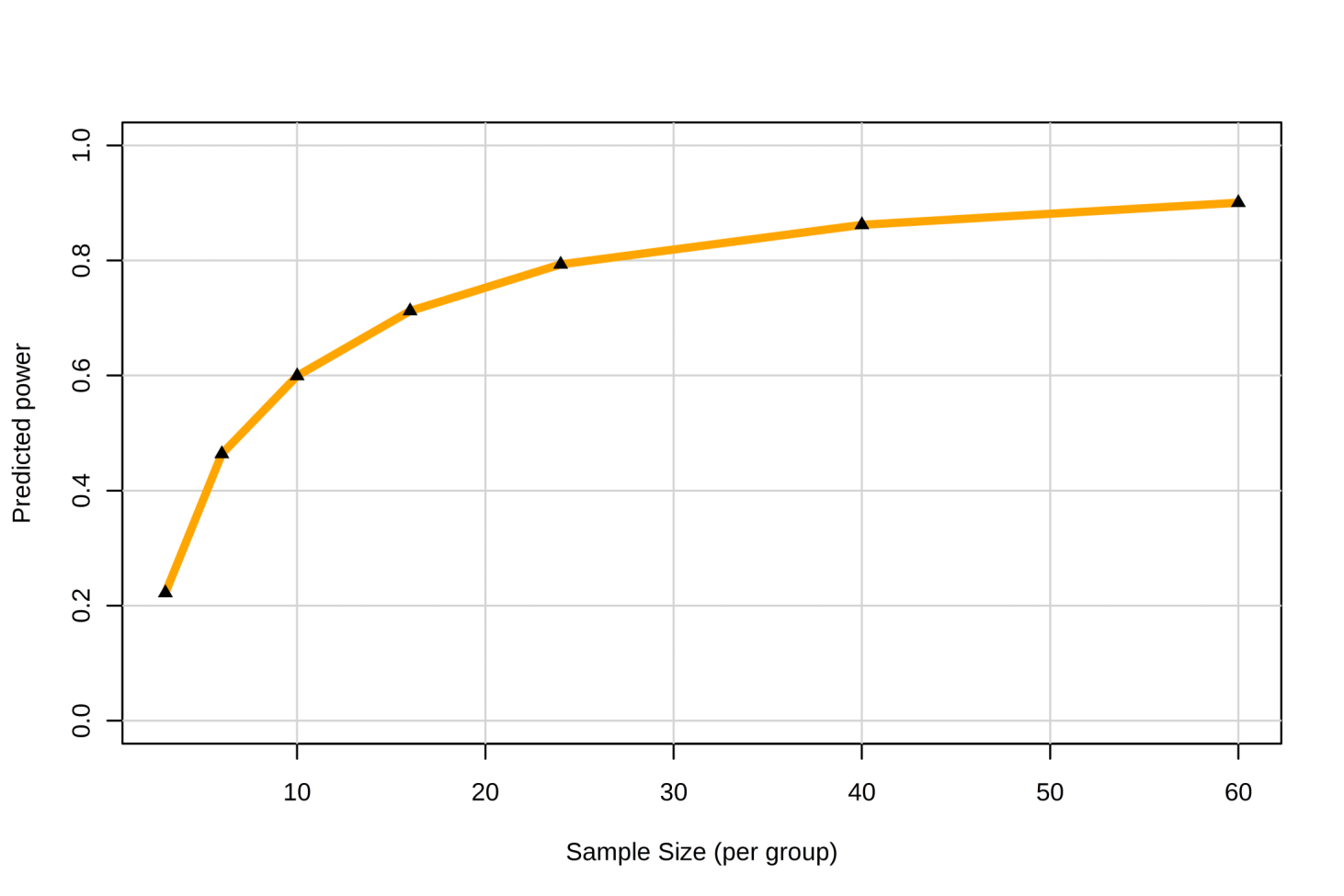


The power analysis suggests that we will have over 80% power to detect discriminating metabolites using approximately 25 subjects in each group.

**1.2. Chemicals**

Acetonitrile and spectroscopic formic acid were purchased from Fisher Scientific (ON, Canada). Deionized ultrapure water was made in-house using Milli-Q System from Millipore Corporation (MA, USA). The ESI low concentration tuning mix and API-TOF reference mass solution containing Hexakisphosphazine (2.5 mM), Purine (5.0 mM) and Ammonium Trifluroacetate (100.00 mM) were obtained from Agilent Technologies (CA, USA).

**1.3. Extraction of plasma metabolites**

Briefly, plasma samples (100 microliters) were mixed with acetonitrile (200 microliters) in 1.5 millilitres Eppendorf tubes. The mixture is then vortexed vigorously for 30 seconds and was spun for 10 minutes (7378 x g at 4ºC). The supernatant was transferred into a new tube and dried under a gentle stream of nitrogen gas. The dried samples were reconstituted in 80% acetonitrile in deionized water (100 microliters). Each plasma sample was extracted in duplicate. The reconstituted samples were placed into a glass insert in an amber glass auto-sampler vial prior to LC-QTOF-MS analysis. A quality control (QC) mixture made of pooled plasma samples were extracted by the same method. This QC mixture was used to ensure the stability of LC -MS system. In order to ensure system stability, the pooled QC mixtures were analyzed in a random manner among all other samples. In addition to running the QC samples with all other plasma samples, six injections of pooled QC sample were carried out in both positive and negative mode before running the plasma samples to validate the extraction and LC-MS method.

**1.4. Liquid chromatography separation**

The metabolites were separated on a 1290 Infinity Agilent HPLC system from Agilent Technologies (CA, USA). The chromatographic separation for processed plasma was performed on a Zorbax Extend-C18 analytical column (2.1 mm × 50 mm I.D., particle size 1.8 µm, Agilent Technologies, USA). Water was used as solvent A and acetonitrile was used as solvent B. All solvents for the LC system contained 0.1 % formic acid. Metabolites were eluted from the analytical column with a gradient of 0, 0.5, 12, 12.5 and 13 min with 30, 30, 100, 100 and 30% of solvent B respectively at a flow rate of 400 microliters/min. The column was immediately re-equilibrated at initial conditions (30% solvent B) for 2 min before injecting the next sample. The column temperature was kept at 55°C.

**1.5. MS analysis**

Mass spectral analysis of eluting peptides from the analytical column was carried out on a 6538 UHD Accurate Q-TOF LC/MS from Agilent Technologies (CA, USA) controlled by MassHunter Workstation Software (v 7.0). All analyses were performed in both positive and negative mode ESI employing a dual ionization source. These two sources will ensure that both polar and non-polar compounds are detected. The mass detection was done using reference ions of m/z 121.050873 and 922.009798 for positive mode and m/z 119.03632 and 1033.9881 for negative mode. The instrument settings were: gas temperature - 300 °C; drying N2 gas flow rate - 11 (litre/min); Nebulizer pressure - 50 psig; fragmentor voltage - 175 V; skimmer voltage – 50 V and OCTRF Vpp voltage - 750 V. The collision energy was applied by setting an appropriate equation having a slope value of 5 and offset value of 2.5. A full range mass scan from 50 to 1700 m/z with an extended dynamic range of 2 GHz standardized at 3200 was applied. Data acquisition rate was maintained at a rate of 2 spectra/second using a time frame of 500 milliseconds/spectra and a transient/spectrum ratio of 4057.

**1.6. Data processing and statistical analysis**

The acquired raw LC/MS data (‘.d files’) was preprocessed using Agilent MassHunter Qualitative Analysis (MHQ, vB.07) and Profinder (v B.06) software. Data processing was done by applying the “Find by Formula” (FBF) algorithm to the Total Ion Chromatogram (TIC) files by querying against the custom database to extract features, satisfying an absolute abundances of more than 5000 counts. The custom database was made by collecting information on numerous metabolites associated with cardiovascular disease from published literature and also by combining reported metabolites in blood from Human Metabolome Database (HMDB). The FBF algorithm operates based on the compound’s monoisotopic mass (in ppm), isotope spacing (in ppm) and isotope distribution (in %). During data processing, the match tolerance limit was set to ± 10 ppm for masses and ± 0.35 minutes for retention times. The parameters were chosen to provide information of the compound based on their isotope pattern, multiple charge states, the formation of dimer and adduct ions (+H, +Na, + K, +NH4 adducts in positive ion mode and -H, -HCOO and -CH3COO adducts in negative ion mode). The collected information summarizing retention time (RT), ion intensity, exact mass and possible chemical relationships (isotopes, adducts, dimers, multiple charge states) was converted into compound exchange format (‘*.cef’) files. The ‘*.cef’ files were then imported to Agilent Mass Profiler Professional (MPP, v12.6) software for further data processing and statistical analysis. A frequency filtration was used to only accept features that were detected in at least one of the four conditions (time intervals). The ion intensities for each spectrum were normalized using a percentile shift algorithm set to 75 and were adjusted to the baseline values of the median of all samples. Repeated measures one way ANOVA (p<0.001) was used to identify metabolite changes over the four time intervals within the same subjects. The ‘Bonferroni FWER’ multiple testing correction method was used to adjust p-values derived from multiple statistical tests and to correct for occurrences of false positives. The identified features satisfying the above conditions were then subjected to a recursive analysis using ‘Batch Recursive Feature Extraction’ algorithm in MPP to generate the final list of potential features. In recursive analysis, the list of features already identified by FBF algorithm was re-extracted once again by searching against the raw data files. The log-transformed metabolite concentrations (non-averaged) of the final entity list with 130 differential metabolites across the four time intervals (0 h ischemic condition (pre angioplasty), 2 h post reperfusion , 24 h post reperfusion and 48 h post reperfusion) were used for sample classification and further statistical analysis. The differential metabolites were classified based on their chemical taxonomy on HMDB and KEEG (*Kyoto* Encyclopedia of Genes and Genomes) databases.

Further statistical analysis and biomarker analysis were done using the MetaboAnalyst software v3.0 (McGill University, Quebec, Canada). In order to visualize the similarities and differences between each of the four time intervals in the plasma metabolome, the unsupervised multivariate pattern recognition technique, principal component analysis (PCA) was first employed on the metabolome data. In the PCA score plot, each point represents an individual sample. The loadings (weights) of the metabolites on the principal components indicate which among the four time points were similar, different or distinct. The supervised 3-dimensional partial least squares-discriminant analysis (PLS-DA) was then employed to maximize difference in metabolic profile between the time intervals. A cross comparison of the samples between different time intervals will help us to understand the early and late response in plasma metabolome in response to I/R injury by identifying the key metabolites involved at each junction. To accomplish this, a one-way analysis of variance (ANOVA) with p-value cut-off of 0.05, followed by Tukey's Honestly Significant Difference (Tukey's HSD) post hoc analysis was performed on the log-transformed metabolite concentrations (non-averaged) of the final entity list with 130differential metabolites. Next, to examine the metabolomic pathways represented by these 130 differential metabolites, a pathway impact analysis was performed using MetPA (Metabolomics Pathway Analysis) tool (<http://metpa.metabolomics.ca>) based on KEGG database. The pathway impact was calculated as the sum of the importance measures of the matched metabolites normalized by the sum of the importance measures of all metabolites in each pathway. The differential metabolites identified from one-way ANOVA served as the input metabolite data set representing each time interval. To investigate the relationship of the 130 differential metabolites in the metabolite entity set, the pair-wise correlations (Pearson correlation coefficient) between the metabolites were calculated using correlation calculator from MetScape software v3.0 (http://metscape.ncibi.org) based on the log normalized intensities of the metabolites. Using group average values, only those metabolites satisfying a Pearson correlation coefficient, |r| >0.9 were considered as highly correlated metabolites (n=37). The MetaboAnalyst v4.0 was used to plot the metabolite-metabolite interaction network plot to visualize and analyze biological relationships between the correlated metabolites. Next, to examine the underlying biochemical pathways reflected by this network correlation, a Metabolite Set Enrichment Analysis (MESA) was done on these correlated metabolites. MSEA is a metabolomic version of the popular GSEA (Gene Set Enrichment Analysis) software. MSEA has its own collection of metabolite set libraries (MetaboAnalyst v3.0 library contains 88 metabolite sets based on normal metabolic pathways). MSEA was implemented using the hypergeometric test to evaluate whether a particular metabolite set is represented more than expected by chance within the given compound list. The pathway significance was determined based on fold enrichment and p-value, by searching against the pathway-associated metabolite sets library. To further investigate the relationship between the plasma metabolites and important clinical factors at baseline (0 h, time-1), correlations were also calculated for all pairs of metabolite-clinical factors using the R statistical package ‘corrplot’. In addition, receiver operating characteristic (ROC) analysis was used to evaluate the diagnostic capability of metabolites which can serve as potential biomarkers. A summary of the metabolomic workflow was provided in **Supplementary Table.1**.

| **Supplementary Table.1 Summary of the metabolomic workflow** | |
| --- | --- |
| **Step 1** | **Data processing** |
| Non-targeted analysis of plasma samples in both ESI positive and ESI negative modes. | |
| The processing of acquired raw LC/MS data (‘.d files’) using MHQ vB.07 and Profinder vB.06 software. | |
| “Find by Formula” (FBF) algorithm was used to extract all detectable compounds. The entity list contained 765 and 670 compounds in ESI+ and ESI- modes, respectively. | |
| ‘Batch Recursive Feature Extraction’ algorithm in MPP v12.6.1 to remove false ± compounds. The entity list was reduced to 69 and 82 compounds in ESI+ and ESI- modes, respectively. | |
| Repeated measures one way ANOVA followed by ‘Bonferroni FWER’ multiple testing correction method in MPP. After adjusting for p-value, the final list contained 130 significantly differential metabolites across all the four time points (p<0.001). | |
| **Step 2** | **Statistical analysis - MetaboAnalyst software v3.0** |
| The supervised and unsupervised multivariate pattern recognition techniques, PCA and PLSDA were employed. | |
| A one-way analysis of variance (ANOVA) with p-value cut-off of 0.05, followed by Tukey's Honestly Significant Difference (Tukey's HSD) post hoc analysis to identify significant metabolites at each junction in the time course. | |
| Pathway impact analysis was performed using MetPA (Metabolomics Pathway Analysis) tool to identify biochemical pathways represented by the differential metabolites across different time points. | |
| **Step 3** | **Correlation network analysis** |
| Pair-wise correlation analysis (Pearson correlation coefficient, \|r\|) to identify potential functional relationships between annotated metabolites  - 37 metabolites were found with \|r\| >0.9 using MetScape software v3.0. | |
| Heat map to visualize the highly correlated metabolite concentration across all the time points. | |
| Metabolite Set Enrichment Analysis (MESA) to examine the underlying biochemical pathways reflected by this correlated metabolites. | |
| The MetaboAnalyst v4.0 was used to plot the metabolite-metabolite interaction network plot using these highly correlated metabolites. | |
| The statistical package ‘corrplot’ was used to plot the metabolite – clinical parameters correlation matrix. | |
| **Step 4** | **Diagnostic performance of metabolites** |
| Three metabolites namely pentadecanoic acid, linoleoyl carnitine and 1-linoleoylglycerophosphocholine were selected to perform the ROC analysis. | |
| Multivariate ROC plot based on random forests classification algorithm. | |

**2. Results**

- 1. **Supplementary Figure.2 The number of metabolites identified in different ionization modes**

From the final list of 130 significantly differential metabolites, 55 metabolites were identified exclusively in positive mode, 64 metabolites were identified exclusively in negative mode and 11 metabolites were identified in both the modes (**Refer Supplementary Table.2 for more detail**).


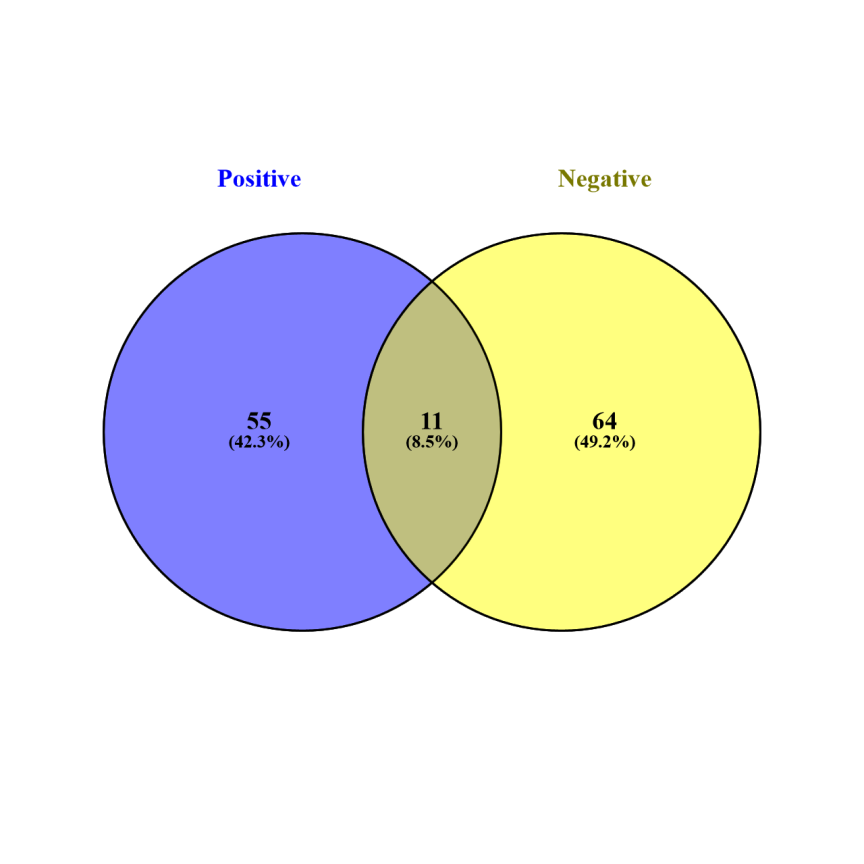


- 1. **Metabolites responsible for early and late response to reperfusion**

The results from cross-comparison among different time intervals employing one-way ANOVA followed by Tukey's HSD identified the key set of metabolites responsible for early and late response to I/R injury. These metabolites were also responsible for the class separation observed with PCA and PLS-DA plots. In total, 11 metabolites were found to be significant between 0 h and 2 h comparison, 96 metabolites were found to be significant between 0 h and 24 h comparison, 107 metabolites were found to be significant between 0 h and 48 h comparison and were 16 metabolites found to be significant between 24 h and 48 h comparison. The numbers of metabolites involved at each time point were shown in below **Supplementary Figure.3 (Refer Supplementary Table.4 for more detail)**


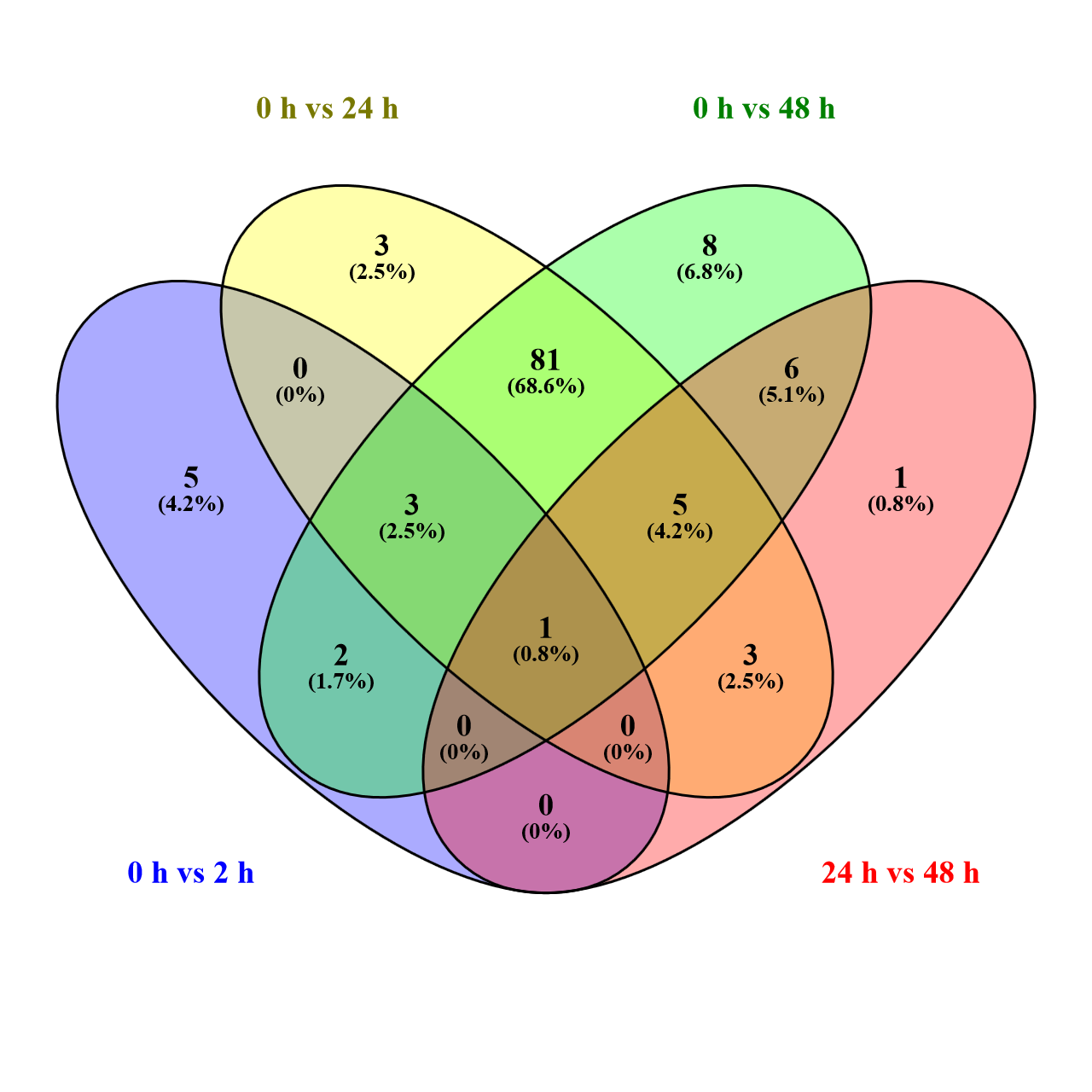


**Supplementary Figure.3 The number of metabolites involved at each time point**

The Venn diagram above depicts the number of metabolites shared among (or unique to) different groups (0 h ischemic condition (pre angioplasty), 2 h post angioplasty, 24 h post angioplasty and 48 h post angioplasty).

- 1. **Correlation between troponin concentration and time to reperfusion**

#
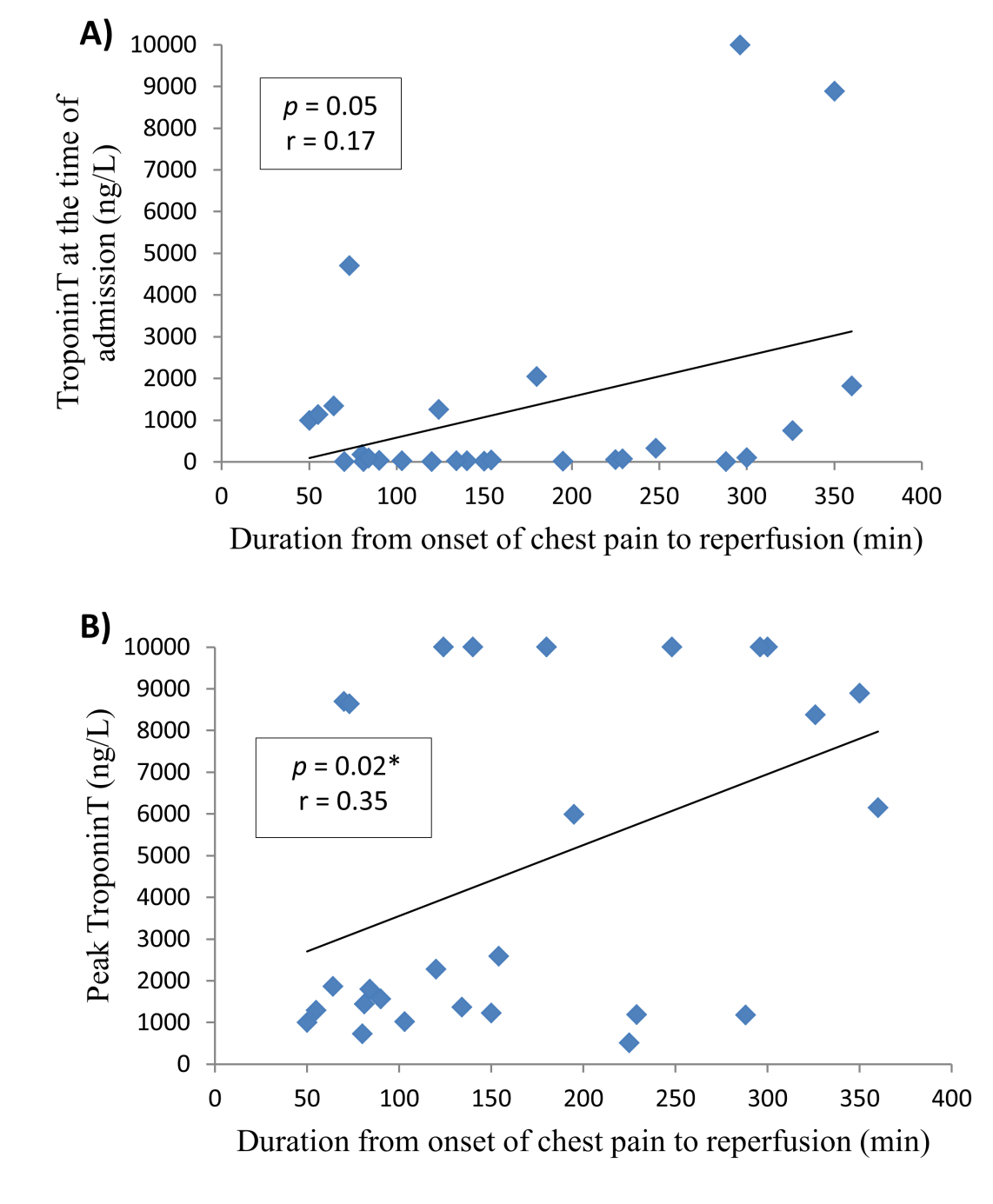


# Supplementary Figure.4 Troponin concentration vs Time to reperfusion

Spearman correlation (r and *p-value*) was calculated to determine the relationship between duration from onset of chest pain to reperfusion and troponin level. From the results, it is evident that time to reperfusion is significantly positively correlated with troponin (both at the time of admission and peak troponin) with correlation coefficient (r) of 0.17 and 0.35 respectively.

- 1. **Supplementary Figure.5 LAD infarct vs RCA infarct vs Circumflex infarct**

**
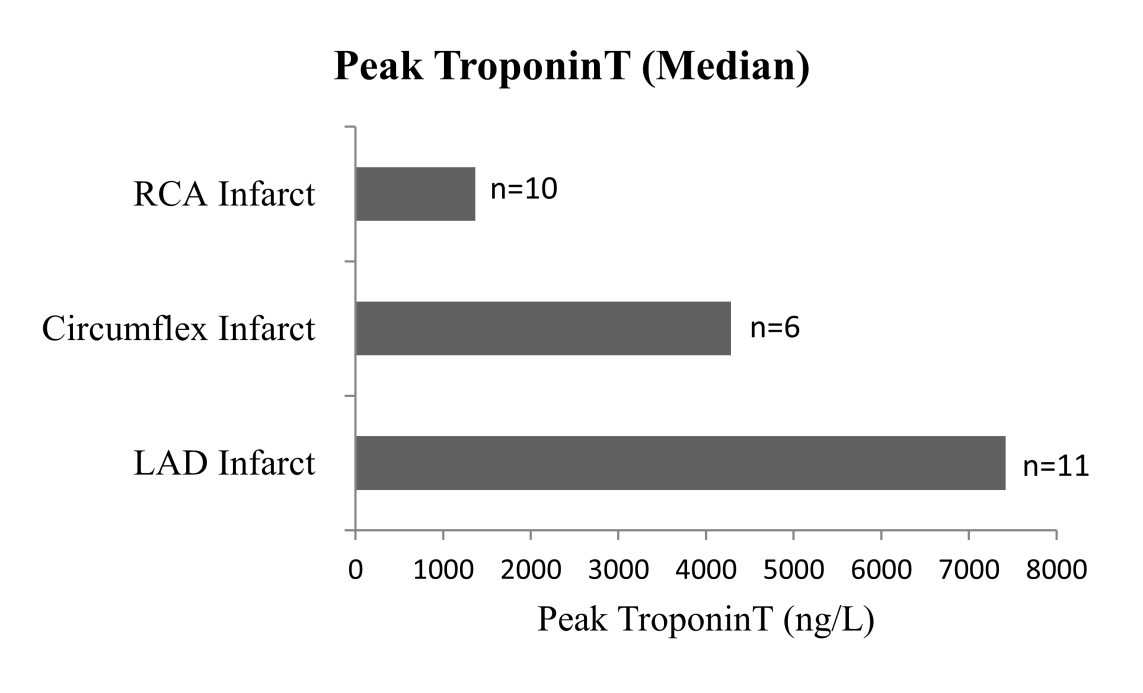
**

We compared the troponin values of LAD infarct with RCA infarct and Circumflex infarct. As expected, the median troponin value is largest for of LAD infarct compared to Circumflex infarct and RCA infarct.

| **Supplementary Table.2 Metabolites identified in different modes** | | | |
| --- | --- | --- | --- |
| **Sl.No** | **55 elements identified exclusively in ESI + mode** | **64 elements identified exclusively in ESI - mode** | **11 common elements in ESI + and ESI -** |
| 1 | 10-Hydroxydihydrosanguinarine | Benzaldehyde | LysoPC 18:3 |
| 2 | Hydroxyisovaleroyl carnitine | 5,8,11-Eicosatrienoic acid | Elaidic acid |
| 3 | Nudifloramide | PA 16:0/16:0 | L Carnitine |
| 4 | Ribitol | PS 18:0/18:2 | Dihomo-linoleate 20:2n6 |
| 5 | 2',3'-Cyclic AMP | LysoPC 20:3 | Linolic acid |
| 6 | Palmitic acid | Dimethyl sulfone | Myo-inositol |
| 7 | 1-palmitoyl-GPC | Sorbitol-6-phosphate | Eicosapentaenoic acid |
| 8 | LysoPE 18:3 | Hydroxypropionylcarnitine | Leucine/Isoleucine |
| 9 | LysoPE 20:4 | Pyroglutamic acid | Palmitoleic acid |
| 10 | 1-methyladenosine | Docosapentaenoic acid | 1-Methylhistamine |
| 11 | L-isoleucyl-L-Proline | 3-Methoxytyramine | 5'-methylthioadenosine |
| 12 | Dehydroascorbic acid | 6-Hydroxygliclazide |  |
| 13 | 3-Octanone | Arachidonic acid |  |
| 14 | LysoPE 22:1 | Thienodihydropyridinium |  |
| 15 | Linoleoyl carnitine | 4-Aminophenol |  |
| 16 | PC 22:4/14:1 | LysoPC 22:4 |  |
| 17 | p-cresol | dCTP |  |
| 18 | Heptadecanoic acid | 15-Keto-13,14-dihydroprostaglandin A2 |  |
| 19 | Isovaleryl carnitine | 3-Hydroxysuberic acid |  |
| 20 | LysoPC 22:6 | D-Limonene |  |
| 21 | Orotic acid | N-Acetylserotonin |  |
| 22 | Xanthine | Mevalonic acid |  |
| 23 | 1-Linoleoylglycerophosphocholine | Docosahexaenoic acid |  |
| 24 | Succinic acid | Glycyl-glycine |  |
| 25 | 2-Nonynoic acid | Leucyl-phenylalanine |  |
| 26 | Ascorbic acid | 11-Ketoetiocholanolone |  |
| 27 | LysoPC 24:0 | N-Acetylaspartylglutamic acid |  |
| 28 | Xanthosine | 3'-O-Methylepicatechin |  |
| 29 | Thromboxane B2 | PG 18:0/18:1 |  |
| 30 | 2-hydroxyglutarate | L-Hexanoylcarnitine |  |
| 31 | Guanine | Thymidine |  |
| 32 | Propanoate | Calcitroic acid |  |
| 33 | Pyruvic acid | N-Acetylornithine |  |
| 34 | Acetate | Equol |  |
| 35 | gamma-Glutamylvaline | 4-Ethylphenol |  |
| 36 | Malonic acid | 3,5-Diiodothyronine |  |
| 37 | O-Phosphothreonine | Oleoyl glycine |  |
| 38 | Pyridoxamine 5'-phosphate | Homocysteine |  |
| 39 | 3-Methoxy-4-Hydroxyphenylglycol sulfate | Acylcarnitine |  |
| 40 | Enterodiol | 1,5-Anhydrosorbitol |  |
| 41 | 5-Hydroxytryptophol | Enkephalin L |  |
| 42 | Dehydrogenated ticlopidine | Hydroxyphenylacetylglycine |  |
| 43 | 3-Hydroxytetradecanedioate | Diacetylspermine |  |
| 44 | Normetanephrine | 1,3,7-Trimethyluric acid |  |
| 45 | Hexanoylglycine | Benzoyl ecgonine |  |
| 46 | PC 16:0/16:0 | Hypoxanthine |  |
| 47 | N-Desmethylpromazine | 12,13-DiHODE |  |
| 48 | Retinylglucuronide | Protoprimulagenin A3 |  |
| 49 | D-Pipecolic acid | O-Desmethylverapamil |  |
| 50 | SM d18:0/16:0 | L-Tryptophan |  |
| 51 | Neopterin | dCMP |  |
| 52 | Aminoacetone | Pregnenolone sulfate |  |
| 53 | Deoxyadenosine | Citrulline |  |
| 54 | PG 18:0/22:4 | Glycodesoxycholate |  |
| 55 | N-Desmethyleletriptan | Didodecylthiobispropanoate |  |
| 56 |  | Phenylacetylglutamine |  |
| 57 |  | Hydrocortisone |  |
| 58 |  | p-Hydroxyphenylacetate |  |
| 59 |  | Oxidized glutathione |  |
| 60 |  | Pentadecanoic acid |  |
| 61 |  | Biliverdin |  |
| 62 |  | Mannitol |  |
| 63 |  | PI 16:0/16:0 |  |
| 64 |  | 4-Hydroxybenzoic acid |  |

| **Supplementary Table.3 Final list of 130 significant metabolites** | | | | | | | |
| --- | --- | --- | --- | --- | --- | --- | --- |
| **Sl.No** | **Compound** | ***P value*** | **HMDB ID** | **m/z** | **Most abundant ionic species** | **RT** | **Pathways** |
| 1 | Benzaldehyde | 5E-15 | HMDB06115 | 151.0404 | (M+HCOO)- | 0.412 | Phenylalanine metabolism |
| 2 | 5,8,11-Eicosatrienoic acid | 0.0004 | HMDB10378 | 305.2483 | (M-H)- | 9.44 | Arachidonic acid metabolism, Linoleic acid metabolism |
| 3 | PA 16:0/16:0 | 6E-08 | HMDB00674 | 647.4623 | (M-H)- | 9.847 | Glycerophospholipid metabolism |
| 4 | PS 18:0/18:2 | 0.0434 | HMDB12380 | 832.5315 | (M+HCOO)- | 7.542 | Glycerophospholipid metabolism |
| 5 | LysoPC 20:3 | 0.03 | HMDB10393 | 590.3463 | (M+HCOO)- | 7.106 | Glycerophospholipid metabolism |
| 6 | Dimethyl sulfone | 0.0157 | HMDB04983 | 187.0111 | (2M-H)- | 0.395 | Sulfur metabolism |
| 7 | Sorbitol-6-phosphate | 7E-17 | HMDB05831 | 261.0378 | (M-H)- | 0.342 | Fructose and mannose metabolism |
| 8 | Hydroxypropionyl carnitine | 3E-05 | HMDB13125 | 525.2693 | (2M+CH3COO)- | 0.393 | Fatty acid metabolism |
| 9 | Pyroglutamic acid | 0.0003 | HMDB00267 | 128.0358 | (M-H)- | 0.337 | Glutathione metabolism |
| 10 | Docosapentaenoic acid | 6E-05 | HMDB06528 | 329.2486 | (M-H)- | 9.19 | Biosynthesis of unsaturated fatty acids, Linoleic acid metabolism |
| 11 | 3-Methoxytyramine | 1E-12 | HMDB00022 | 379.1863 | (2M+HCOO)- | 9.856 | Tyrosine metabolism |
| 12 | 6-Hydroxygliclazide | 1E-06 | HMDB14028 | 398.1398 | (M+CH3COO)- | 9.009 | Gliclazide Pathway |
| 13 | Arachidonic acid | 0.0058 | HMDB01043 | 303.2332 | (M-H)- | 8.931 | Arachidonic acid metabolism, Linoleic acid metabolism |
| 14 | Thienodihydropyridinium | 2E-22 | HMDB13924 | 321.059 | (M+CH3COO)- | 0.355 | NA |
| 15 | 4-Aminophenol | 0.0003 | HMDB01169 | 263.1033 | (2M+HCOO)- | 0.351 | Aminobenzoate degradation, Microbial metabolism in diverse environments |
| 16 | LysoPC 22:4 | 0.0062 | HMDB10401 | 616.3621 | (M+HCOO)- | 7.697 | Glycerophospholipid metabolism |
| 17 | dCTP | 0.0032 | HMDB00998 | 978.9779 | (2M+HCOO)- | 0.323 | Pyrimidine metabolism |
| 18 | 15-Keto-13,14-dihydroprostaglandin A2 | 2E-06 | HMDB01244 | 667.4251 | (2M-H)- | 9.843 | NA |
| 19 | 3-Hydroxysuberic acid | 3E-06 | HMDB00325 | 379.1627 | (2M-H)- | 9.016 | Fatty acid metabolism |
| 20 | D-Limonene | 0.0001 | HMDB03375 | 331.2648 | (2M+CH3COO)- | 9.862 | Limonene and pinene degradation, Biosynthesis of terpenoids and steroids |
| 21 | N-Acetylserotonin | 0.0002 | HMDB01238 | 263.1033 | (M+HCOO)- | 0.351 | Tryptophan metabolism |
| 22 | Mevalonic acid | 1E-09 | HMDB00227 | 355.161 | (2M+CH3COO)- | 9.564 | Terpenoid backbone biosynthesis, Biosynthesis of terpenoids and steroids, Ferroptosis |
| 23 | Docosahexaenoic acid | 0.0063 | HMDB02183 | 327.233 | (M-H)- | 8.814 | Biosynthesis of unsaturated fatty acids, Linoleic acid metabolism |
| 24 | Glycyl-glycine | 0.0012 | HMDB11733 | 263.0994 | (2M-H)- | 0.354 | Glycine metabolism |
| 25 | LysoPC 18:3 | 0.0009 | HMDB10388 | 562.3151 | (M+HCOO)- | 5.843 | Glycerophospholipidmetabolism |
| 26 | Leucyl-phenylalanine | 0.0001 | HMDB13243 | 277.1536 | (M-H)- | 5.6 | Phenylalanine metabolism |
| 27 | 11-Ketoetiocholanolone | 1E-05 | HMDB06031 | 667.4251 | (2M+CH3COO)- | 9.843 | NA |
| 28 | N-Acetylaspartyl glutamic acid | 7E-26 | HMDB01067 | 653.1788 | (2M+HCOO)- | 3.086 | Alanine, aspartate and glutamate metabolism |
| 29 | 3'-O-Methylepicatechin | 1E-31 | HMDB29175 | 653.1906 | (2M+HCOO)- | 3.092 | Flavonoid biosynthesis |
| 30 | PG 18:0/18:1 | 3E-08 | HMDB10603 | 836.5805 | (M+CH3COO)- | 9.198 | Glycerophospholipid metabolism |
| 31 | L-Hexanoylcarnitine | 0.0256 | HMDB00756 | 577.3737 | (2M+CH3COO)- | 10.617 | Fatty acid metabolism |
| 32 | Thymidine | 2E-08 | HMDB00273 | 241.0836 | (M-H)- | 2.469 | Pyrimidine metabolism |
| 33 | Calcitroic acid | 2E-07 | HMDB06472 | 433.2601 | (M+CH3COO)- | 9.893 | NA |
| 34 | N-Acetylornithine | 2E-11 | HMDB03357 | 407.2144 | (2M+CH3COO)- | 0.518 | Arginine and proline metabolism, Biosynthesis of amino acids |
| 35 | Equol | 3E-12 | HMDB02209 | 241.0873 | (M-H)- | 2.445 | NA |
| 36 | 4-Ethylphenol | 0.0007 | HMDB29306 | 121.0658 | (M-H)- | 0.501 | Bisphenol degradation |
| 37 | 3,5-Diiodothyronine | 6E-14 | HMDB00582 | 1108.8 | (2M+CH3COO)- | 8.807 | Thyroid hormone synthesis |
| 38 | Oleoyl glycine | 0.0153 | HMDB13631 | 338.2707 | (M-H)- | 8.365 | NA |
| 39 | Homocysteine | 3E-10 | HMDB00742 | 194.0501 | (M+CH3COO)- | 0.382 | Homocysteine Degradation, Betaine Metabolism, Methionine Metabolism |
| 40 | Acylcarnitine | 2E-08 | HMDB01185 | 444.1451 | (M+HCOO)- | 8.628 | Arginine and proline metabolism, Cysteine and methionine metabolism, Biosynthesis of amino acids |
| 41 | 1,5-Anhydrosorbitol | 6E-12 | HMDB02712 | 327.1295 | (2M-H)- | 8.216 | NA |
| 42 | Enkephalin L | 3E-14 | HMDB01045 | 1169.5576 | (2M+CH3COO)- | 0.326 | Neuroactive ligand-receptor interaction |
| 43 | Hydroxyphenylacetylglycine | 9E-29 | HMDB00735 | 477.1525 | (2M+CH3COO)- | 4.111 | Tyrosine metabolism |
| 44 | Diacetylspermine | 0.0001 | HMDB02172 | 345.249 | (M+CH3COO)- | 7.636 | NA |
| 45 | 1,3,7-Trimethyluric acid | 2E-15 | HMDB02123 | 255.0743 | (M+HCOO)- | 0.409 | Caffeine metabolism |
| 46 | Benzoyl ecgonine | 1E-06 | HMDB41836 | 637.2768 | (2M+CH3COO)- | 6.592 | NA |
| 47 | Hypoxanthine | 0.0308 | HMDB00157 | 181.0373 | (M+HCOO)- | 0.343 | Purine metabolism, Caffeine metabolism |
| 48 | 12,13-DiHODE | 2E-09 | HMDB10201 | 669.4582 | (2M+HCOO)- | 10.89 | Linoleic acid metabolism |
| 49 | Protoprimulagenin A3 | 1E-14 | HMDB33157 | 1147.5594 | (M+HCOO)- | 0.297 | NA |
| 50 | O-Desmethylverapamil | 0.0014 | HMDB13961 | 499.2817 | (M+CH3COO)- | 5.411 | (Drug metabolite) |
| 51 | ^a^L-Tryptophan^8,43,44^ | 1E-13 | HMDB00929 | 263.1033 | (M+CH3COO)- | 0.351 | Glycine, serine and threonine metabolism, Phenylalanine, tyrosine and tryptophan biosynthesis, Tryptophan metabolism, Biosynthesis of amino acids |
| 52 | ^a^dCMP^45^ | 9E-11 | HMDB01202 | 352.0566 | (M+HCOO)- | 0.413 | Pyrimidine metabolism |
| 53 | ^a^Pregnenolone sulfate^46^ | 0.0002 | HMDB00774 | 395.1894 | (M-H)- | 3.184 | Steroid hormone biosynthesis |
| 54 | ^a^Citrulline^8^ | 0.0007 | HMDB00904 | 395.1894 | (2M+HCOO)- | 3.184 | Arginine biosynthesis, Biosynthesis of amino acids |
| 55 | ^a^Glycodesoxycholate^7^ | 1E-05 | HMDB00631 | 448.3072 | (M-H)- | 3.874 | NA |
| 56 | ^a^Didodecyl thiobispropanoate^44^ | 7E-05 | HMDB40172 | 1087.8188 | (2M+CH3COO)- | 9.202 | NA |
| 57 | ^a^Phenylacetylglutamine^7^ | 8E-10 | HMDB06344 | 263.1038 | (M-H)- | 0.35 | Phenylalanine metabolism |
| 58 | ^a^Hydrocortisone^43^ | 2E-21 | HMDB14879 | 407.2076 | (M+HCOO)- | 0.519 | Bile secretion, Steroid hormone biosynthesis, |
| 59 | ^a^p-Hydroxy phenylacetate^47^ | 2E-24 | HMDB00020 | 151.0402 | (M-H)- | 0.392 | Phenylalanine metabolism, Tyrosine metabolism |
| 60 | ^a^Oxidized glutathione^8^ | 5E-14 | HMDB03337 | 611.1449 | (M-H)- | 3.627 | Glutathione metabolism, Ferroptosis |
| 61 | ^a^Pentadecanoic acid^8^ | 1E-05 | HMDB00826 | 241.2174 | (M-H)- | 8.903 | Fatty acid metabolism |
| 62 | ^a^Biliverdin^46^ | 2E-21 | HMDB01008 | 581.2407 | (M-H)- | 10.53 | Porphyrin and chlorophyll metabolism |
| 63 | ^a^Mannitol^8^ | 2E-21 | HMDB00765 | 241.093 | (M+CH3COO)- | 2.415 | Fructose and mannose metabolism |
| 64 | ^a^PI 16:0/16:0^46^ | 2E-21 | HMDB09778 | 855.5254 | (M+HCOO)- | 8.738 | Glycerophospholipid metabolism |
| 65 | ^a^4-Hydroxybenzoic acid^8^ | 4E-30 | HMDB00500 | 137.0244 | (M-H)- | 0.525 | Phenylalanine metabolism, Ubiquinone and other terpenoid-quinone biosynthesis |
| 66 | ^a^10-Hydroxy dihydrosanguinarine^48^ | 1E-06 | NA | 372.084 | (M+Na)+ | 2.484 | Isoquinoline alkaloid biosynthesis |
| 67 | ^a^Elaidic acid^49^ | 3E-13 | HMDB00573 | 283.263 | (M+H)+ | 10.011 | Fatty acid biosynthesis, Biosynthesis of unsaturated fatty acids |
| 68 | ^a^Hydroxyisovaleroyl carnitine^8^ | 4E-26 | HMDB62555 | 523.323 | (2M+H)+ | 10.449 | Fatty acid metabolism |
| 69 | ^a^L Carnitine^8,48^ | 2E-33 | HMDB00062 | 162.1118 | (M+H)+ | 0.331 | Bile secretion |
| 70 | ^a^dihomo-linoleate (20:2n6)^8^ | 0.0005 | HMDB61864 | 331.2629 | (M+Na)+ | 9.374 | Linoleic acid metabolism |
| 71 | ^a^ Linoleic acid^7,8,49^ | 1E-07 | HMDB00673 | 281.2473 | (M+H)+ | 9.181 | Linoleic acid metabolism |
| 72 | ^a^Nudifloramide^46^ | 0.0004 | HMDB04193 | 153.0656 | (M+H)+ | 0.371 | Nicotinate and nicotinamide metabolism |
| 73 | ^a^Ribitol^47^ | 0.012 | HMDB00508 | 153.0746 | (M+H)+ | 0.353 | Pentose and glucuronate interconversions, Riboflavin metabolism |
| 74 | ^a^Myo-inositol^8^ | 0.0085 | HMDB00211 | 203.0526 | (M+Na)+ | 0.324 | Ascorbate and aldarate metabolism, Galactose metabolism |
| 75 | ^a^2',3'-Cyclic AMP^43,50^ | 7E-30 | HMDB11616 | 347.0872 | (M+NH4)+ | 0.424 | Purine metabolism |
| 76 | ^a^Palmitic acid^44,46^ | 3E-11 | HMDB00220 | 257.2473 | (M+H)+ | 9.751 | Fatty acid biosynthesis, Biosynthesis of unsaturated fatty acids |
| 77 | ^a^1-palmitoyl glycerophosphocholine^8^ | 2E-06 | HMDB62541 | 519.3264 | (M+Na)+ | 5.924 | Glycerophospholipid metabolism |
| 78 | ^a^Eicosapentaenoic acid^51^ | 8E-14 | HMDB01999 | 303.2314 | (M+H)+ | 8.383 | Biosynthesis of unsaturated fatty acids |
| 79 | ^a^LysoPE 18:3^7^ | 4E-15 | HMDB11509 | 476.2761 | (M+H)+ | 5.449 | Glycerophospholipidmetabolism |
| 80 | ^a^Leucine/Isoleucine^8,43^ | 1E-13 | HMDB00687 | 132.1017 | (M+H)+ | 0.341 | Valine, leucine and isoleucine biosynthesis, Biosynthesis of amino acids |
| 81 | ^a^LysoPE 20:4^7^ | 0.0027 | HMDB11517 | 502.2927 | (M+H)+ | 6.145 | Glycerophospholipid metabolism |
| 82 | ^a^1-methyladenosine^51^ | 0.0013 | HMDB03331 | 299.1446 | (M+NH4)+ | 0.38 | NA |
| 83 | ^a^L-isoleucyl-L-Proline^43^ | 0.0006 | HMDB11174 | 229.154 | (M+H)+ | 0.334 | NA |
| 84 | ^a^Dehydroascorbic acid^47^ | 5E-05 | HMDB01264 | 366.0682 | (2M+NH4)+ | 0.399 | Ascorbate and aldarate metabolism, Glutathione metabolism |
| 85 | ^a^3-Octanone^7^ | 4E-11 | HMDB31295 | 257.2475 | (2M+H)+ | 9.759 | NA |
| 86 | ^a^LysoPE 22:1^7^ | 0.0062 | HMDB11521 | 536.3697 | (M+H)+ | 8.544 | Glycerophospholipid metabolism |
| 87 | ^a^Linoleoyl carnitine^52^ | 4E-08 | HMDB06469 | 424.3415 | (M+H)+ | 5.661 | Fatty acid metabolism |
| 88 | ^a^PC 22:4/14:1^46^ | 6E-07 | HMDB08624 | 780.5525 | (M+H)+ | 9.758 | Glycerophospholipid metabolism, Arachidonic acid metabolism, Linoleic acid metabolism |
| 89 | ^a^p-cresol^47^ | 0.0191 | HMDB01858 | 126.0914 | (M+NH4)+ | 0.354 | Toluene degradation |
| 90 | ^a^Heptadecanoic acid^8^ | 1E-24 | HMDB02259 | 271.2623 | (M+H)+ | 10.367 | Biosynthesis of unsaturated fatty acids |
| 91 | ^a^Isovaleryl carnitine^52^ | 6E-12 | HMDB00688 | 268.1544 | (M+Na)+ | 0.337 | Leucine metabolism, Fatty acid metabolism |
| 92 | Palmitoleic acid | 0.0196 | HMDB03229 | 255.2315 | (M+H)+ | 8.769 | Fatty acid biosynthesis |
| 93 | ^a^LysoPC 22:6^7^ | 2E-06 | HMDB10404 | 568.3398 | (M+H)+ | 6.597 | Glycerophospholipidmetabolism |
| 94 | ^a^Orotic acid^45^ | 1E-05 | HMDB00226 | 174.0524 | (M+NH4)+ | 0.362 | Pyrimidine metabolism |
| 95 | ^a^Xanthine^8,45^ | 5E-30 | HMDB00292 | 153.0415 | (M+H)+ | 0.363 | Purine metabolism, Caffeine metabolism |
| 96 | ^a^1-Linoleoyl glycerophosphocholine^51^ | 8E-23 | HMDB61692 | 1091.7001 | (2M+Na)+ | 7.252 | Glycerophospholipid metabolism |
| 97 | ^a^Succinic acid^8,44,45,53^ | 5E-30 | HMDB00254 | 141.016 | (M+Na)+ | 0.472 | Phenylalanine metabolism, Tyrosine metabolism, Citrate cycle (TCA cycle), Butanoate metabolism, Alanine, aspartate and glutamate metabolism, Oxidative phosphorylation |
| 98 | ^a^2-Nonynoic acid^7^ | 5E-30 | HMDB0032442 | 172.1333 | (M+NH4)+ | 0.337 | NA |
| 99 | ^a^Ascorbic acid^8^ | 0.0004 | HMDB00044 | 194.0658 | (M+NH4)+ | 0.695 | Glutathione metabolism, Ascorbate and aldarate metabolism |
| 100 | ^a^5'-methylthioadenosine^46^ | 5E-30 | HMDB01173 | 336.0508 | (M+K)+ | 0.994 | Cysteine and methionine metabolism |
| 101 | ^a^LysoPC 24:0^7^ | 8E-06 | HMDB10405 | 646.4209 | (M+K)+ | 9.842 | Glycerophospholipid metabolism |
| 102 | ^a^1-Methylhistamine^45^ | 5E-30 | HMDB00898 | 126.0994 | (M+H)+ | 10.023 | Histidine metabolism |
| 103 | ^a^Xanthosine^53^ | 3E-05 | HMDB00299 | 285.0811 | (M+H)+ | 0.695 | Purine metabolism, Caffeine metabolism |
| 104 | ^a^Thromboxane B2^43^ | 0.0002 | HMDB03252 | 741.4745 | (2M+H)+ | 9.99 | Arachidonic acid metabolism, Bile secretion |
| 105 | ^a^2-hydroxyglutarate^8,44^ | 5E-30 | HMDB59655 | 193.0351 | (M+HCOO)+ | 1.563 | Butanoate metabolism |
| 106 | ^a^Guanine^44^ | 5E-30 | HMDB00132 | 152.0562 | (M+H)+ | 0.909 | Purine metabolism |
| 107 | Propanoate | 5E-30 | NA | 132.1016 | (2M+NH4)+ | 0.332 | Citrate cycle (TCA cycle) |
| 108 | ^a^Pyruvic acid^8^ | 0.0004 | HMDB00243 | 201.0247 | (2M+Na)+ | 0.674 | Phenylalanine metabolism, Tyrosine metabolism, Citrate cycle (TCA cycle), Vitamin B6 metabolism, |
| 109 | ^a^Acetate^44^ | 5E-30 | NA | 141.016 | (2M+Na)+ | 0.472 | Citrate cycle (TCA cycle) |
| 110 | ^a^gamma-Glutamylvaline^48^ | 8E-10 | HMDB11172 | 515.2332 | (2M+Na)+ | 5.933 | NA |
| 111 | ^a^Malonic acid^43^ | 5E-30 | HMDB00691 | 267.0371 | (2M+CH3COO)+ | 0.384 | Pyrimidine metabolism |
| 112 | O-Phosphothreonine | 0.0002 | HMDB11185 | 200.0337 | (M+H)+ | 0.419 | Porphyrin and chlorophyll metabolism |
| 113 | Pyridoxamine 5'-phosphate | 3E-07 | HMDB01555 | 249.0614 | (M+H)+ | 0.327 | Vitamin B6 metabolism |
| 114 | 3-Methoxy-4-Hydroxy phenylglycolsulfate | 8E-16 | HMDB03332 | 265.0352 | (M+H)+ | 0.329 | norepinephrine metabolism |
| 115 | Enterodiol | 2E-13 | HMDB05056 | 627.2916 | (2M+Na)+ | 7.019 | NA |
| 116 | 5-Hydroxytryptophol | 0.0011 | HMDB01855 | 200.068 | (M+Na)+ | 0.311 | Serotonin degradation |
| 117 | Dehydrogenated ticlopidine | 0.0253 | HMDB13926 | 262.0451 | (M+H)+ | 0.399 | (Drug metabolite) |
| 118 | 3-Hydroxytetra decanedioate | 4E-07 | HMDB00394 | 571.3446 | (2M+Na)+ | 6.601 | NA |
| 119 | Normetanephrine | 5E-05 | HMDB00819 | 367.1877 | (2M+H)+ | 8.388 | Tyrosine metabolism |
| 120 | Hexanoylglycine | 0.0002 | HMDB00701 | 347.2207 | (2M+H)+ | 1.013 | Fatty acid metabolism |
| 121 | PC 16:0/16:0 | 0.0418 | HMDB00564 | 734.5682 | (M+H)+ | 11.442 | Glycerophospholipid metabolism |
| 122 | N-Desmethylpromazine | 3E-07 | HMDB13939 | 541.2479 | (2M+H)+ | 5.518 | (Drug metabolite) |
| 123 | Retinylglucuronide | 0.0001 | HMDB10340 | 480.2972 | (M+NH4)+ | 6.157 | (Drug metabolite) |
| 124 | D-Pipecolic acid | 0.0233 | HMDB05960 | 130.0862 | (M+H)+ | 0.333 | Lysine degradation |
| 125 | SM d18:0/16:0 | 0.0062 | HMDB10168 | 744.557 | (M+K)+ | 10.683 | Sphingolipid metabolism |
| 126 | Neopterin | 1E-22 | HMDB00845 | 524.1952 | (2M+NH4)+ | 4.445 | Folate biosynthesis |
| 127 | Aminoacetone | 4E-05 | HMDB02134 | 169.0932 | (2M+Na)+ | 0.379 | Glycine, serine and threonine metabolism |
| 128 | Deoxyadenosine | 2E-20 | HMDB00101 | 525.1936 | (2M+Na)+ | 4.429 | Purine metabolism |
| 129 | PG 18:0/22:4 | 0.0013 | HMDB10611 | 849.5645 | (M+Na)+ | 9.955 | Glycerophospholipid metabolism |
| 130 | N-Desmethyleletriptan | 8E-12 | HMDB13919 | 369.1627 | (M+H)+ | 8.373 | (Drug metabolite) |
| ^a^ - The compounds already reported in published literature known to be associated with either ischemia, myocardial infarction or other forms of CAD including non-obstructive coronary atherosclerosis, stable angina pectoris or unstable angina pectoris; *P Value* - *P-values* after ‘Bonferroni FWER’ multiple testing correction; HMDB ID - Human Metabolome Database ID; m/z – mass to charge ratio of the most abundant ion; RT – retention time | | | | | | | |
